# Optimized RNA interference therapeutics combined with interleukin-2 mRNA for treating hepatitis B virus infection

**DOI:** 10.1038/s41392-024-01871-8

**Published:** 2024-06-21

**Authors:** Wenjing Zai, Min Yang, Kuan Jiang, Juan Guan, Huijing Wang, Kongying Hu, Chao Huang, Jieliang Chen, Wei Fu, Changyou Zhan, Zhenghong Yuan

**Affiliations:** 1 Key Laboratory of Medical Molecular Virology (MOE/NHC/CAMS), Research Unit of Cure of Chronic Hepatitis B Virus Infection (CAMS), Shanghai Frontiers Science Center of Pathogenic Microbes and Infection, School of Basic Medical Sciences, Shanghai Medical College, Fudan University, Shanghai, P. R. China; 2 Department of Pharmacology, School of Basic Medical Sciences, Fudan University, Shanghai, P. R. China; 3 Shanghai Engineering Research Center for Synthetic Immunology, Fudan University, Shanghai, P. R. China; 4 Eye Institute and Department of Ophthamology, Eye and ENT Hospital, Fudan University, Shanghai, P. R. China; 5 Pharmacy Department of Huashan Hospital, Fudan University, Shanghai, P. R. China; 6 Institute of Pediatric Translational Medicine, Shanghai Institute for Pediatric Congenital Heart Disease, Shanghai Children’s Medical Center, School of Medicine, Shanghai Jiao Tong University, Shanghai, China; 7 Shanghai Institute of Infectious Disease and Biosecurity, Shanghai, P. R. China

**Keywords:** Nucleic-acid therapeutics, Drug development

## Abstract

This study aimed to develop a pan-genotypic and multifunctional small interfering RNA (siRNA) against hepatitis B virus (HBV) with an efficient delivery system for treating chronic hepatitis B (CHB), and explore combined RNA interference (RNAi) and immune modulatory modalities for better viral control. Twenty synthetic siRNAs targeting consensus motifs distributed across the whole HBV genome were designed and evaluated. The lipid nanoparticle (LNP) formulation was optimized by adopting HO-PEG_2000_-DMG lipid and modifying the molar ratio of traditional polyethylene glycol (PEG) lipid in LNP prescriptions. The efficacy and safety of this formulation in delivering siHBV (tLNP/siHBV) along with the mouse IL-2 (mIL-2) mRNA (tLNP/siHBVIL2) were evaluated in the rAAV-HBV1.3 mouse model. A siRNA combination (terms “siHBV”) with a genotypic coverage of 98.55% was selected, chemically modified, and encapsulated within an optimized LNP (tLNP) of high efficacy and security to fabricate a therapeutic formulation for CHB. The results revealed that tLNP/siHBV significantly reduced the expression of viral antigens and DNA (up to 3log_10_ reduction; vs PBS) in dose- and time-dependent manners at single-dose or multi-dose frequencies, with satisfactory safety profiles. Further studies showed that tLNP/siHBVIL2 enables additive antigenic and immune control of the virus, via introducing potent HBsAg clearance through RNAi and triggering strong HBV-specific CD4^+^ and CD8^+^ T cell responses by expressed mIL-2 protein. By adopting tLNP as nucleic acid nanocarriers, the co-delivery of siHBV and mIL-2 mRNA enables synergistic antigenic and immune control of HBV, thus offering a promising translational therapeutic strategy for treating CHB.

## Introduction

Hepatitis B virus (HBV) infection can lead to chronic hepatitis B (CHB), liver fibrosis, cirrhosis, and hepatocellular carcinoma (HCC); however, therapeutic options are limited.^1^ Nucleot(s)ide analogues efficiently suppress viral replication but fail to reduce viral antigen levels, PEGylated interferons possess both direct antiviral and immunomodulating effects, whereas the efficacy is moderate, and response rate in clinical patients is low. The HBV genome is a compact 3.2 kb circular, partially double-stranded, relaxed circular DNA (rcDNA). Once entering the nucleus, rcDNA is repaired by endogenous enzymes into covalently closed circular DNA (cccDNA), which encodes four overlapping open reading frames (ORFs), including the core antigen (HBcAg), surface antigen (HBsAg), e antigen (HBeAg), and X protein (HBx).^2^ All HBV transcripts have a common 3′ end and share the same polyadenylation signal (PAS). Besides, replication-incompetent HBV linear fragments can integrate into the host genome (intHBV), serving as the predominant source of HBsAg in HBeAg-negative patients.^3–5^ High-circulating HBV antigen levels, especially HBsAg, contribute to immune tolerance and viral persistence.^6,7^ Since complete cure (clearance of cccDNA and intHBV) is unattainable, the evolving treatment goal for CHB is to achieve HBsAg seroclearance with therapy of finite duration, that is, “functional cure”.^8–10^

RNA interference (RNAi) technology has shown promises for treating genetic disease and virus-infection diseases. It is also considered as an attractive therapeutic modality to achieve functional cure of CHB by inducing antigen inhibition, viraemia reduction, and cccDNA silence.^11–13^ siRNA-based therapies can also relieve high viral antigen-induced immune tolerance, offering opportunity to gain immune control of the viruses by subsequent immune-stimulation. However, designing functional small interfering RNAs (siRNAs) against complicated viruses like HBV is challenging considering the extraordinarily high genetic diversity among the 10 different genotypes (A to J).^14–16^ siRNA designed for targeting conserved regions among HBV genotypes is achievable by computational prediction and is anticipated to resist potential viral mutational escape, whereas siRNA targeting the common 3′-end sequences shared by cccDNA-derived transcripts may loss targets on intHBV-derived transcripts.^16–18^ Further studies using siRNA triggers designed to address both cccDNA- and intHBV-driven synthesis are expected to surpass those of previous agents in terms of efficacy and functionality.

The clinical application of RNAi therapeutics is hindered by challenges associated with liver-targeted delivery systems. With advantages of newly developed GalNAc ((N-acetylgalactosamine) technology and lipid nanoparticles for liver-specific delivery, siRNA-based drugs such as Partisiran [Onpattro®] and Inclisiran [Leqvio®] are clinically available. Recently, ionizable cationic lipid nanoparticles (LNPs) were designed for the efficient delivery of therapeutic nucleic acids in vivo.^19^ LNPs are typically composed of ionizable cationic lipids, cholesterol, polyethylene glycol (PEG) lipids, and auxiliary lipids. PEG lipids provide a neutral hydrophilic exterior, stabilize the particle, and prevent rapid clearance in the circulation. However, there is also “PEG dilemma”. PEG ratio affects the performance of LNP in vivo. Despite the weak immunogenicity of PEG, a certain proportion of humans may develop low levels of PEG-specific antibodies, leading to accelerated clearance of PEGylated nanomedicines and reduced efficacy.^20,21^ The terminal PEGylation group influences PEG immunogenicity, with hydroxy-PEG being less immunogenic than methoxy-PEG.^22^ Moreover, our group recently uncovered that hydroxy-PEG has weaker antigenicity than methoxy-PEG. Hydroxy-PEG can avoid the binding of pre-existing anti-PEG antibodies in human blood, thereby reducing complement activation, which allows LNPs to escape antibody recognition and rapid clearance.^23^

Although RNAi- and antibody-mediated HBsAg clearance may withdraw immune dysfunction by reducing the viral burden, they cannot activate viral-specific T cells to realize long-term viral control.^24,25^ Only tiny minority of patients produce antibodies against HBsAg in the clinic, and viral antigens recover after drug withdrawal.^26–28^ Recently, pre-clinical and clinical studies have been investigating novel combination strategies for attaining improved therapeutic outcomes, such as combined RNAi therapeutics with therapeutic vaccines, interferons, and anti-HBs antibodies.^29–31^ Studies indicated that IL-2 is a critical mediator for proper viral antigen presentation and activation and differentiation of virus-specific CD4^+^ and CD8^+^ T cells.^32–35^ Sequential low-dose IL-2 along with IFN-α can increase the frequency and restore the function of HBV-specific CD8^+^ T cell responses in patients,^36^ indicating the potential of constructing viral-specific immune control to complement RNAi. We propose that concurrent suppression of the viral load by RNAi and enhancement of host immunity by IL-2 may be sufficient to break immune tolerance and reconstruct antiviral immunity.

In the present study, we screened and chemically modified a pan-genotypic and multifunctional siRNA combination (siHBV) against all forms of cccDNA- and intHBV-derived transcripts, and validated its efficacy and safety in multiple cell culture and murine models. An optimized LNP platform (tLNP) with low antigenicity and high efficacy was utilized to define the role of siHBV in controlling HBV transcription and replication and to evaluate its safety profile. By utilizing the feasibility of tLNP to encapsulate different forms of nucleic acids, co-delivery of siHBV and mouse IL-2 (mIL-2) mRNA within a single tLNP formulation was applied to obtain both antigenic and immune control of the virus. We anticipate that tLNP-based siHBV and mIL-2 mRNA co-delivery may provide a feasible approach for the treatment of CHB.

## Materials and methods

### siRNA design

To design highly conservative siRNA against HBV, a collection of 11,185 HBV genome sequences were downloaded from the Hepatitis Virus Database (HVDB) website (http://s2as02.genes.nig.ac.jp/index.html) and analyzed for highly conserved motifs via motif-based sequence analysis tools (MEME Suite 5.4.1) (https://meme-suite.org/meme/tools/meme). HBV typical sequences were put into to professional siRNA design websites, including siDirect, siDESIGN Center, DSIR et al. Twenty siRNAs targeting HBV conserved motifs were selected distributing throughout the HBV whole genome. The criteria used for siRNA design were as follows: 1. Accordance with the basic design principle provided by online software; 2. Have limited homology with any known sequence in the human, mouse and rat genomes. Unmodified and chemically modified siRNA used in this study were then synthesized by RiboBio (Guangzhou, China) and GenePharma (Shanghai, China) for experiments.

We defined “conservation” as the percentage of sequence entries out of the 11,185 HBV sequences that showed perfect identity with the 19/2-18-mer.

### Cell lines and transfection

HepG2-HBV^EGFP^ cell line was constructed in-house as previous reported.^37,38^ In brief, pSBbi-HBV^EGFP^ was created by fusion of the *loxP* sites flanking monomeric linear HBV sequence (GenBank accession no. V01460.1) with the EGFP sequence and the splicing acceptor sequence inserted into the core region in the HBV genome to the pSBbi vector (Addgene plasmid #60525). The pSBbi-HBV^EGFP^ and pCMV-SB100 plasmids were then co-transfected into HepG2 cells to pick neomycin-resistance colonies and to construct stable cell lines.

HepG2, HepAD38, HepG2-NTCP, and HepG2-HBV^EGFP^ cells were maintained in DMEM supplemented with 10% fetal bovine serum (FBS) and 1% penicillin-streptomycin. PLC/PRF/5 cells were maintained in RPMI-1640 medium supplemented with 10% FBS and 1% penicillin-streptomycin. Cells were seeded in 96-, 48-, or 24- well plates, then treated with indicated doses of siRNA. Transfection was carried out using Lipofectamine RNAiMAX transfection reagents (Invitrogen, USA) according to the manufacturer’s protocol or using LNPs. Supernatants and cells were collected at 72 h post-transfection.

Pan-genotypic antiviral activity was conducted in HepG2 cells transfected with plasmid DNA harboring 1.3 × overlength sequence of HBV genotype A, B, C, and D isolates. HepG2 cells were transfected with HBV genotype A-D encoding plasmids, then treated with varying doses of siRNA. Culture supernatants were collected at 72 h post-transfection and assayed for HBsAg and HBeAg levels using ELISA (Autobio Diagnostics Co., Ltd., China).

siRNA was transfected with RNAiMAX at the concentration of 0, 6.25, 12.5, 25, 50, and 100 nM, respectively. For HBV antigen and mRNA examination, the cells and supernatants were harvested separately at 72 h post-transfection.

### Animal experiments

C57BL/6 mice (5–6 weeks old) were purchased from GemPharmatech company (Jiangsu, China). Animals were maintained under specific pathogen-free conditions in Fudan University Laboratory Animal Center or in the ABSL-2 animal facility at the Key Laboratory of Medical Molecular Virology (MOE/NHC/CAMS), School of Basic Medical Sciences, Shanghai Medical College, Fudan University. All procedures involving experimental animals were performed according to the protocols approved by the Animal Ethics Committee of School of Basic Medical Science, Fudan University (approval No. 20211115-004).

### Efficacy of tLNP/siHBV in chronic HBV carrier mouse

The rAAV-HBV1.3 mouse model^39^ was employed to evaluate the gene silencing efficiency of siHBV encapsulating tLNP (terms “tLNP/siHBV”). In brief, male C57BL/6 mice were inoculated with 2.5 × 10^10^ vector genomes (v.g.)/mice of rAAV-HBV1.3 virus carrying a 1.3 × overlength HBV genome (ayw, Five Plus, Beijing, China). Animals were bled one day before start of treatment and divided into groups to obtain similar HBsAg and HBeAg levels. Six groups of mice (eight animals per group) were dosed with 1× phosphate-buffered saline (PBS), tLNP/siNC, tLNP/siHBV, and ETV, respectively. Both siNC and siHBV were modified by 2′-O-methylation to improve their stability. The dose of tLNP/siNC was 1 mg/kg. The doses of tLNP/siHBV were 0.1, 0.3 and 1 mg/kg, respectively. A single dose of tLNP/siHBV formula was intravenously injected into mice at indicated doses. Blood samples were collected weekly during the experiments. Liver specimens were collected for histological and molecular analyses.

For multidose experiments, tLNP/siHBV were weekly (five doses in total) or biweekly (three doses in total) injected into the mice. The serum samples were collected weekly, and were applied for HBsAg, HBeAg and HBV DNA analysis according to the manufacturer’s instructions. The mice were sacrificed at the end of experiment, and tissue samples were collected for further detection.

### Virologic analysis

Viral titers in the serum were quantified with HBV-specific primers and probes using commercial kits (Sansure Biotech, Changsha, China) with a standard curve for absolute quantification. HBsAg, HBeAg, anti-HBs and anti-HBc levels were quantified via ELISA analysis using commercial kits (AutoBio Diagnostics Co., Ltd., Zhengzhou, China), respectively.

As for HBV-derived mRNA, such as pgRNA and total RNAs, cell or liver RNA were extracted and reverse transcribed into cDNA using PrimeScript^TM^ RT reagent Kit with gDNA Eraser (Takara, Japan), and real-time quantitative PCR was performed using SYBR^®^ Premix Ex Taq^TM^ (Tli RNaseH Plus), Bulk (Takara, Japan). HBV-specific primers were used as follows: HBV pgRNA (forward: 5′-GCCTTAGAGTCTCCTGAGCA-3′; reverse: 5′-GAGGGAGTTCTTCTTCTAGG-3′) and the HBV total RNA (forward: 5′-GCTTTCACTTTCTCGCCAAC-3′; reverse: 5′-GAGTTCCGCAGTATGGATCG-3′). The expression of human or mouse housekeeping genes (GAPDH, β-Actin) were used for normalization.

As for intrahepatic HBsAg levels, liver sections were homogenized with PBS, centrifuged, and applied for HBsAg determination and protein quantification using commercial kits. Intrahepatic cccDNA, core particle DNA and total RNA were extracted as we previous reported,^40^ then applied for southern blot, northern blot and real-time quantitative PCR analysis with specific primers.

### Histology and immunohistochemistry

Organs were collected and fixed in 10% formaldehyde, embedded in paraffin, and then sectioned for H&E staining. Histopathologic evaluation was performed by an experienced liver pathologist in a blinded manner. For immunochemistry, anti-CD8 (Servicebio, Wuhan, China), anti-CD4 (Servicebio, Wuhan, China) and anti-HBc (Long Island Antibody, Shanghai, China) antibodies were used.

### Flow cytometry analysis

For flow cytometry analysis, isolated cells were suspended in FACS buffer. Cell-surface staining was performed using anti-mouse CD3, anti-mouse CD8 (Alexa Fluor 700, eBioscience) and anti-mouse CD4 (eFluor™ 506, eBioscience) antibodies. Dead cells were excluded from analysis by fixable viability dye eF780 staining. Intracellular cytokine staining was performed using indicated antibody after fixation and permeabilization. Data were collected using Attune flow cytometer (Thermo Fisher, USA) and analyzed using FlowJo software version 10 (Tree Star, Ashland, OR). H-2Kb tetramer conjugated with HBV-derived peptides S109 (VWLSVIWM) and C93 (MGLKFRQL) were purchased from Creative Biosciences (Guangzhou, China), and were subjected for detecting HBsAg-specific and core-specific CD4^+^ and CD8^+^ T cells.

### Statistical analysis

The data were expressed as the means ± standard deviations (SDs) or means ± standard error of means (SEMs). One-way or two-way analysis of variance (ANOVA) was applied for multiple comparisons. Significance was defined as *P < 0.05, **P < 0.01, ***P < 0.001, and ****P < 0.0001.

## Results

### Narrow regions screening across HBV whole-genome for multifunctional, highly-conserved siRNA triggers

**Fig. 1:**
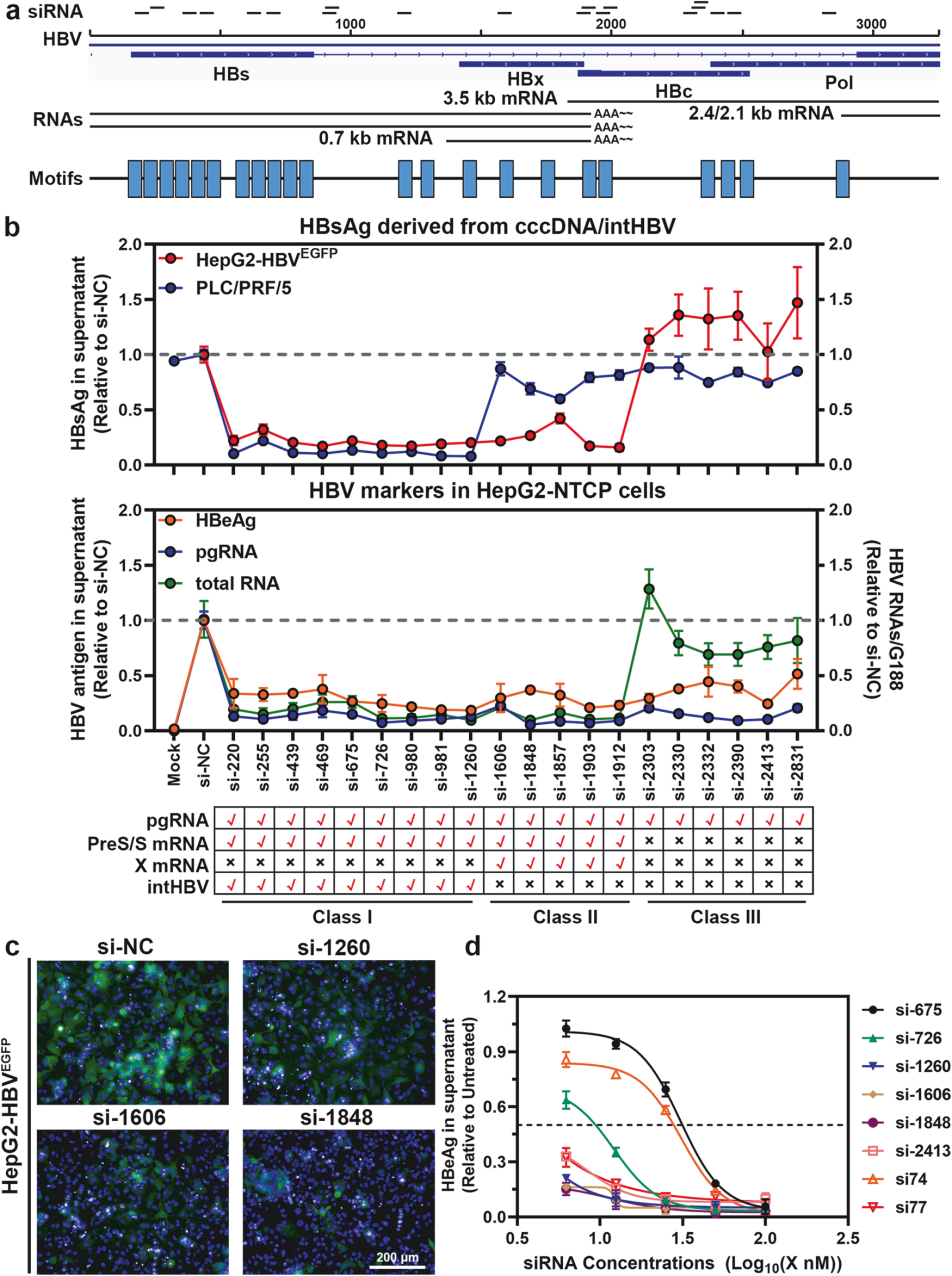
Genome-wide scanning of conservative and efficient anti-HBV siRNA triggers. **a** The structure of HBV RNAs and the location of siRNA candidates. The siRNA candidates are shown as short lines. The locations of four HBV ORFs (HBs, HBc, HBx and Pol) and the relative transcripts (3.5 kb, 2.4 kb, 2.1 kb, and 0.7 kb) are indicated. The distribution of 22 conserved motifs in HBV genome are also indicated. **b** Twenty siRNA candidates were transfected by RNAiMAX transfection reagents and screened at the dose of 50 nM in HepG2-HBV^EGFP^ cells (HBsAg derived from recombination cccDNA. The reporting cell line was constructed in-house) and PLC/PRF/5 cells (HBsAg derived from HBV integrates. The cell line carrying natural HBV integrates was derived from clinical patents). The supernatants were collected at 72 h post-transfection, and the HBsAg levels in the culture medium were determined by commercial ELISA kits and was normalized to that of cells treated with a negative control siRNA (siNC). HepG2-NTCP (NTCP receptor-overexpressing HepG2 cell line that supports HBV infection) cells were infected with HBV at a multiplicity of infection (MOI) of 200 for 3 days, then transfected with siRNA candidates at the dose of 50 nM using transfection reagents (RNAiMAX). HBeAg expression in the culture medium was analyzed at 72 h post-transfection, and the intracellular pgRNA and total RNA levels were detected with the corresponding primers. **c** The inhibitory efficacy of siRNAs on EGFP expression levels in HepG2-HBV^EGFP^ cells were visualized by high content imaging (HCI) and screening (HCS) assay using Operetta CLS (PerkinElmer). Scale bar indicated 200 μm. **d** HepG2-NTCP cells were infected with HBV at the MOI of 200, then treated with serial dilutions of siRNA (0, 6.25, 12.5, 25, 50, and 100 nM) using RNAiMAX transfection reagent. HBeAg expression levels in the supernatants were determined by ELISA kits, and the IC_50_ values were calculated. The si74 and si77 (two siRNA triggers from ARC-520) were used as positive controls. Data were shown as means ± SDs (*n* = 3 ~ 4)

We sequenced the HBV genome using the Hepatitis Virus Database (HVDB) and motif-based sequence analysis tools (MEME) (Supplementary Fig. 1). Twenty-two conserved motifs were identified (Supplementary Table 1) and 20 siRNA sequences targeting the conserved motifs distributed across the entire HBV genome were synthesized for further examination (Fig. 1a). These siRNAs were subsequently categorized into three groups based on their theoretical target mRNAs, as shown in Supplementary Table 2 and Fig. 1b (class I: targets pgRNA, PreS/S RNAs; class II: targets pgRNA, PreS/S, X RNAs; class III: targets only pgRNA).

The interference efficiency of the siRNA candidates was assessed using the following cell models: PLC/PRF/5 is a clinical hepatocarcinoma-derived cell line that carries natural HBV integration with varying breakpoints and secrets HBsAg from intHBV.^41,42^ The HepG2-HBV^EGFP^ cell line, which is established in-house, contains recombinant cccDNA with an EGFP reporter gene and secrets HBsAg from cccDNA.^37,38^ As shown in Fig. 1b, most siRNA candidates (class I and class II) significantly reduced cccDNA-derived HBsAg levels in HepG2-HBV^EGFP^ cells, except for class III siRNAs (si-2303-si-2831). Conversely, class II siRNAs (si-1606~si-1912) lost their ability to target intHBV-derived HBsAg transcripts in PLC/PRF/5 cells. Similarly, all candidate siRNAs efficiently reduced the secretion of HBeAg and intracellular pgRNA levels in HepG2-NTCP (HBV receptor transduced cell line) infection model compared to the negative control siRNA (si-NC), whereas class III siRNAs failed to reduce HBV total RNA levels. These results suggested that only narrow regions across the HBV genome were reasonable for designing multifunctional siRNA reagents that target all cccDNA- and intHBV-derived mRNAs with a single siRNA.

The conservativity of the siRNA candidates was calculated (Supplementary Table 3). The efficacy of siRNA candidates was confirmed using 1.3× overlength HBV plasmids of different genotypes (A/B/C/D) and HBV-infected primary human hepatocytes (PHHs) (Supplementary Fig. 2). The interference efficiency was also confirmed using the EGFP reporter protein (Fig. 1c), and compared with two siRNA triggers from ARC-520 (si74 and si77) (Fig. 1d). siRNAs with high interference potency across different genotypes were selected for further analysis.

### Combined siRNA triggers possessed pan-genotypic and multifunctional anti-HBV efficacy

**Fig. 2:**
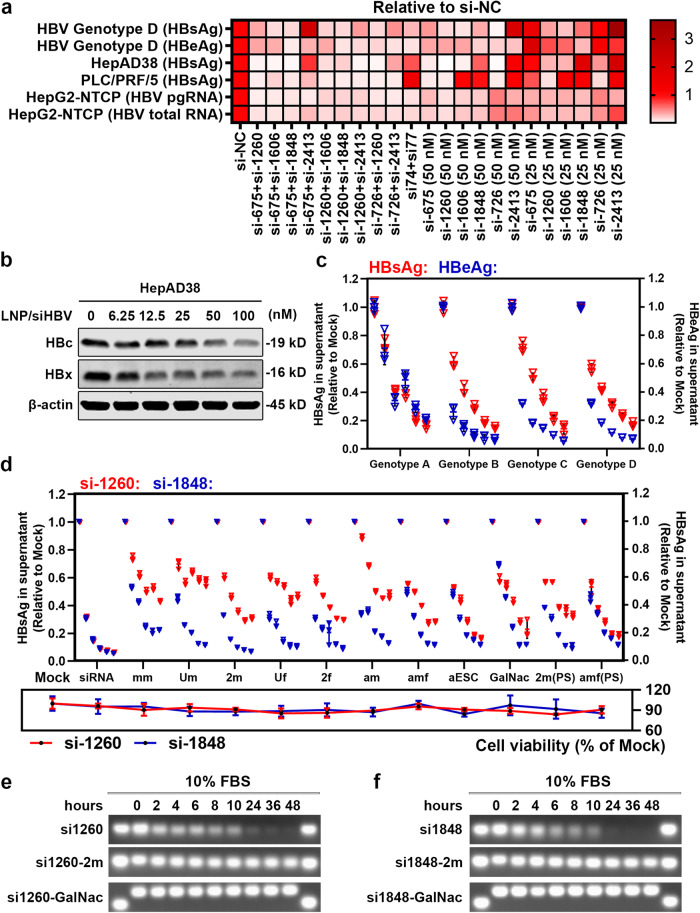
Screening of pan-genotypic and multifunctional siRNA combinations and chemical modifications. **a** HepG2 cells transfected with pHBV1.3 plasmids (Genotype D), HepAD38 (HepG2 cells that stably integrated with a 1.1× overlength HBV genome to support HBV replication) cells, PLC/PRF/5 and HepG2-NTCP cells that infected with HBV at the MOI of 200 were treated with siRNA at the doses of 50 nM or 25 nM, or siRNA combinations at the dose of 25 nM each, using RNAiMAX transfection reagents. The HBsAg and HBeAg levels in the supernatants were determined via ELISA kits at 72 h post-transfection, the intracellular HBV pre-genomic RNA (pgRNA) and HBV total RNA were determined via real-time quantitative PCR (RT-qPCR). **b** HepAD38 cells were treated with varying doses of LNP-formulated siRNA (0, 6.25, 12.5, 25, 50, and 100 nM) and then the expression levels of intracellular HBx and HBc were determined by western blot analysis. **c** HepG2 cells were transfected with pHBV1.3 plasmids of genotypes A, B, C, and D. One day following pHBV1.3 transfection, the cells were transfected with different doses of LNP-formulated siRNA (0, 6.25, 12.5, 25, 50, and 100 nM), then the supernatants were collected and applied for HBsAg and HBeAg detection at 72 h post-transfection. **d** HepG2-HBV^EGFP^ cells were transduced with Ad-Cre and transfected with serial dilutions of si-1260 or si-1848 (0, 6.25, 12.5, 25, 50, and 100 nM) with varying chemical modifications as shown in Supplementary Table 5. HBsAg expression in the supernatant was determined 3 days after transfection. Cell viability was detected by cell counting kit-8 (CCK-8) analysis at 2 days post-transfection. Data were shown as means ± SDs (*n* = 3). **e** The unmodified and modified si-1260 and (**f**) si-1848 were incubated with 10% fetal bovine serum (FBS) in PBS for indicated periods, and the stability of siRNA was evaluated using gel-red electrophoresis and photographed with imaging system

To obtain pan-genotypic and multifunctional siRNA triggers against all HBV-derived transcripts, different siRNA combinations were evaluated for their conservation (Supplementary Table 4) and anti-HBV activities. As shown in Fig. 2a, the combination of si-1260 and si-1848 (terms “siHBV” in the following text) provided means of pan-genotypic functionality (19-mer coverage of 98.55%) against all forms of HBV transcripts, thus was selected for further analysis. In addition, siHBV-encapsulating LNPs effectively reduced intracellular HBc and HBx protein levels in a dose-dependent manner (Fig. 2b). Potent and equipotent inhibition of viral antigens against different HBV genotypes was also observed, confirming pan-genotypic anti-HBV activity (Fig. 2c).

Co-injection of unmodified dissociative siHBV with the prcccDNA/pCMV-Cre plasmid (4 μg each) system by high volume hydrodynamic injection (HDI) significantly reduced HBV antigen levels (1.4–2.3 log_10_ IU/mL reduction of HBsAg, vs Mock), whereas the antigen levels recovered significantly at 4 days post-injection, indicating the short half-lives of un-stabilized and unformulated siRNAs (Supplementary Fig. 3a–c). Naked siRNAs are subject to degradation (especially by the innate immune response triggered in vivo or degradation by nucleases). Chemical modification may dramatically increase in vivo stability and abrogate the undesirable immunostimulatory properties of siRNA.^43^ Thus, the siHBV was chemically stabilized at desired sites with 2′-O-methyl group (2′-OMe), 2′-Fluro (2′-F), and the sulfate bond modification in the backbone, the incorporation of which could reduce immunogenicity, improve stability and extend half-life of siRNAs (Supplementary Table 5). These siRNAs were screened for biological activity, cytotoxicity, and serum stability (Fig. 2d–f and Supplementary Fig. 3d, e). Both siRNA triggers with partial or full 2′-OMe and 2′-F modifications efficiently preserved the knockdown bioactivity, and significantly improved serum stability after incubation with 10% FBS at 37 °C for indicated durations, with limited cellular toxicity. Since si-1260 was sensitive but si-1848 was well-tolerated to chemical modifications, the siRNAs with satisfactory bioactivity, minimal chemical modifications, and superior stability (si1260-2m and si1848-2m) were selected for further evaluation.

### Optimal LNP formulation enables efficient delivery of siHBV

**Fig. 3:**
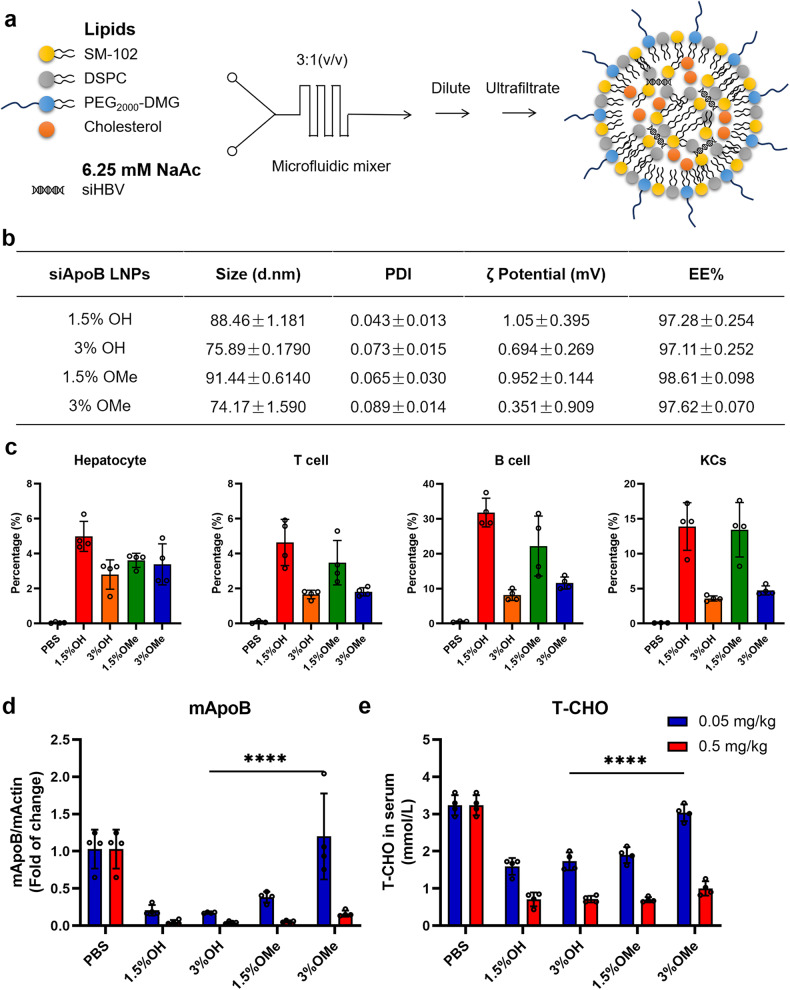
Optimization of low antigenic and hepatocyte-targeted LNP-based siRNA delivery system. **a** Schematics of the preparation procedures of LNPs. **b** Different formulations of LNPs (abbreviated as 1.5% OH, 3% OH, 1.5% OMe and 3% OMe) were prepared, and the sizes, polydispersity index (PDI), ζ potential and encapsulation efficiency (EE%) were analyzed. **c** C57BL/6 mice were intravenously injected with different formulations of LNPs/Cy5-siRNA at the dose of 0.5 mg/kg, 4 h post-injection, the hepatocytes and liver-associated lymphocytes were isolated, and the percentage of Cy5-positive hepatocytes, T cells, B cells and Kupffer cells (KCs) were determined by flow cytometry analysis. Data were shown as means ± SDs (*n* = 4). **d** C57BL/6 mice were intravenously injected with different formulations of LNPs/siApoB at the doses of 0.5 mg/kg and 0.05 mg/kg, mice were sacrificed at 72 h post-injection. Relative *ApoB* mRNA expression levels in the liver of mice (compared with *β-actin* mRNA levels) were determined at 72 h post-injection, and (**e**) the sera total cholesterol (T-CHO) levels were detected via commercial kits. Data were analyzed using two-way ANOVA with Sidak multiple comparison correction, and shown as means ± SDs (*n* = 4). *P < 0.05, **P < 0.01, ***P < 0.001, and ****P < 0.0001

LNPs were selected as the nanocarriers for efficient and tissue-specific delivery of siHBV in vivo. Considering the antigenicity of methoxyl PEG against pre-existing anti-PEG antibodies (data not shown here), HO-PEG_2000_-DMG (with a hydroxyl terminus) lipids at varying molar ratios were explored to replace mPEG_2000_-DMG (with a methoxyl terminus) which is commonly used in approved LNP formulations. The diameters of the nanoparticles ranged from 60 to 100 nm, and 3% molar ratio of PEGylated lipids (both HO-PEG and mPEG) resulted in a smaller mean value of ~75 nm. For all formulations, the encapsulation efficiency (EE%) of siRNA was > 97% (Fig. 3a, b). Many studies reported that the unwanted uptake of LNP by the immune system is a major cause of LNP toxicity and limits its efficacy.^23,44^ Thus, the in vivo performance of LNPs/siRNA was studied in mice using Cy5-labeled siRNA (Cy5-siRNA) as a model siRNA. Biodistribution of LNPs/Cy5-siRNA in different organs of mice was determined at 4 h after injection (at a dose of 0.5 mg/kg), showing reduced lung, spleen and kidney distribution of 3% PEG_2000_-DMG-based LNPs, comparing to that of 1.5% PEG_2000_-DMG-based LNPs, whereas the biodistribution of LNPs in the liver was comparable (Supplementary Fig. 4a–c). The 3% PEG_2000_-DMG-based LNPs also showed better LNP uptake when co-cultured with Huh7 cells for 4 h in vitro (Supplementary Fig. 4d). Hepatocytes and liver-associated lymphocytes were collected 4 h after injection, and the results indicated that 3% PEG_2000_-DMG-based LNPs significantly reduced immune cell uptake with comparable hepatocyte uptake of Cy5-siRNA (Fig. 3c and Supplementary Fig. 5a–d). Meanwhile, the 3% OH-PEG_2000_-DMG-based LNP displayed higher hepatocyte uptake than that of 3% mPEG_2000_-DMG-based LNP at the dose of 0.05 mg/kg (Supplementary Fig. 5e). The splenocytes were collected, showing reduced off-target immune cell uptake as well (at the dose of 0.5 mg/kg) (Supplementary Fig. 6).

Apolipoprotein B (ApoB) is the major apolipoprotein of low-density lipoproteins (LDL) that specifically expressed in hepatocytes, and siRNA targeting ApoB (siApoB) was utilized as another model siRNA to evaluate hepatocyte-targeted delivery and knockdown efficiency of siRNA-encapsulating nanoparticles. Briefly, siApoB encapsulating LNPs were intravenously injected into mice at doses of 0.05 and 0.5 mg/kg, respectively. The liver was dissected 72 h after injection and analyzed for intrahepatic *ApoB* mRNA levels. In addition, intrahepatic and seral total cholesterol (T-CHO) and total triglycerides (TG) levels were determined. The results showed that mice treated with LNPs/siApoB exhibited significantly reduced *ApoB* mRNA expression and circulating T-CHO levels (Fig. 3d, e). Specifically, 3% HO-PEG_2000_-DMG-based LNP showed higher knockdown efficiency than that of 3% mPEG_2000_-DMG-based LNP at the dose of 0.05 mg/kg. Subsequently, the 3% HO-PEG_2000_-DMG SM-102-based LNP (terms “tLNP”) was selected for further evaluation of liver targeted efficacy and knockdown activity in primary mouse hepatocytes (Supplementary Fig. 7a) and in mice. The tLNP/siApoB formulation was intravenously injected into mice at doses of 1, 0.5, 0.1,0.05, 0.01, 0.005 and 0.001 mg/kg. Blood and liver samples were harvested 72 h after injection. The results showed that the formulation potently reduced intrahepatic *ApoB* mRNA levels in a dose-dependent manner, with an ED_50_ value of 0.02303 mg/kg (Supplementary Fig. 7b), the effects of which were better than that of 3% mPEG_2000_-DMG-based LNP (with an ED_50_ value of 0.06127 mg/kg) (Supplementary Fig. 7g). Additionally, intrahepatic TG and T-CHO levels elevated dose-dependently, while seral T-CHO levels reduced accordingly (Supplementary Fig. 7c–f).

### tLNP/siHBV demonstrates satisfactory biosafety

**Fig. 4:**
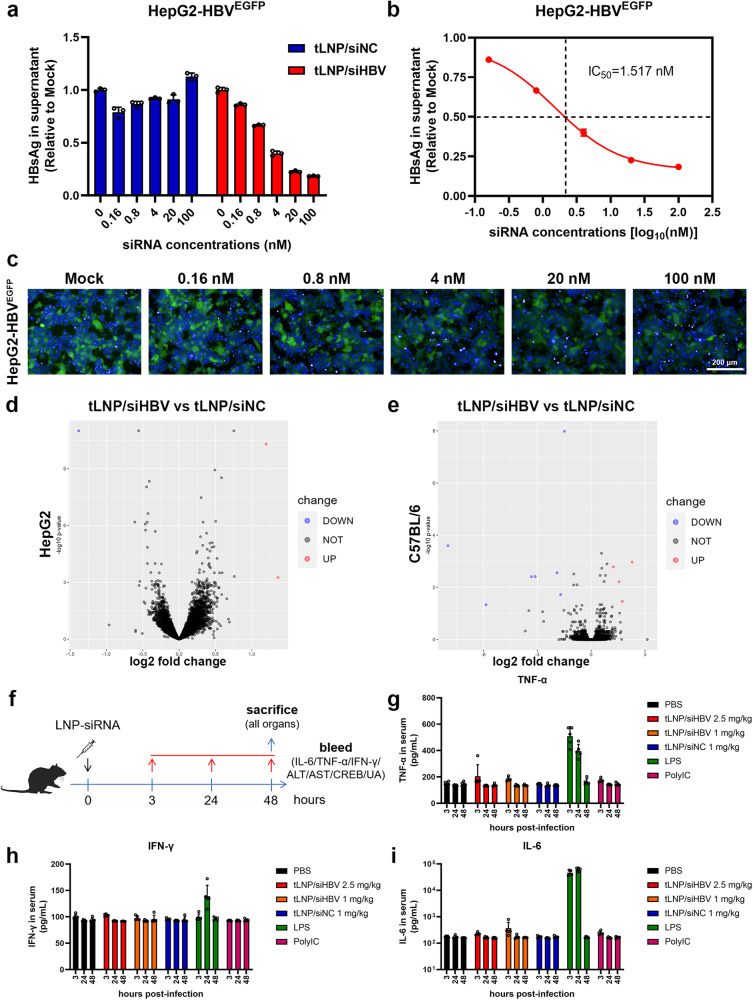
The efficacy and safety of tLNP/siHBV formulations. **a** HepG2-HBV^EGFP^ cells were transduced with Ad-Cre at the MOI of 8 and then transfected for 72 h with varying concentrations of modified siHBV, and HBsAg expression was determined via commercial kits. Data were shown as means ± SDs (*n* = 3). **b** The IC_50_ was calculated by GraphPad Prism software (Version 9.00, San Diego, California, USA). **c** The EGFP expression levels of the treated HepG2-HBV^EGFP^ cells were visualized with high content imaging (HCI) system. Scale bar indicated 200 μm. **d** Global transcriptional profiling of HepG2 cells following treatment with tLNP/siNC and tLNP/siHBV at the dose of 50 nM for 16 h. The volcano plots depicting gene expression changes between tLNP/siHBV and tLNP/siNC treated cells (*n* = 3). **e** Transcriptional dysregulation of mice liver after intravenous injection of tLNP/siHBV (1 mg/kg) or tLNP/siNC (1 mg/kg) for 48 h (*n* = 3). The volcano plots depicting gene expression changes between tLNP/siHBV and tLNP/siNC were shown. **f** Treatment and sampling schedule of toxicity study in mice. 1 mg/kg and 2.5 mg/kg doses of tLNP/siHBV formulations were intravenously injected into C57BL/6 mice. Mice that treated with PBS and 1 mg/kg of tLNP/siNC (i.v.) were utilized as negative controls, and the lipopolysaccharides (LPS) (5 mg/kg, s.c.) and polyI:C treated mice (10 mg/kg, s.c.) were included as positive controls in the assay. **g** Cytokine concentrations in the serum including TNF-α, (**h**) IFN-γ, and (**i**) IL-6 were determined via commercial ELISA kits at indicated time points. Data were shown as means ± SDs (*n* = 5)

The tLNP was explored for the delivery of chemically modified siHBV to evaluate their antiviral efficacy and biosafety in vitro and in vivo. The tLNP was prepared (see Supplementary Methods), and the stability of siRNA encapsulating tLNP was evaluated at −20 °C, 4 °C, or 25 °C for a storage period ranging from 1 week to 4 weeks. The results presented in Supplementary Fig. 8a, b suggested that tLNP/siRNA was stable under the tested conditions. The in vitro analysis revealed that tLNP-formulated siHBV (tLNP/siHBV) suppressed HBsAg and EGFP in a dose-dependent manner in HepG2-HBV^EGFP^ cells, with an IC_50_ value of 1.517 nM (Fig. 4a–c). The dose-dependent anti-HBV effects of tLNP/siHBV were also validated in stable HBV-inducing cell lines, HepAD38 and HepDE19 cells, as well as HBV integrative cell line, PLC/PRF/5 cells (Supplementary Fig. 9).

The RNAi-mediated, miRNA-like off-target effects of siRNAs are major concerns for their clinical application. Chemical modification-related toxicities arising from non-specific binding to cellular proteins and immune recognition of exogenous nucleic acids are major drivers of siRNA hepatotoxicity.^45,46^ We performed global transcriptome profiling of HepG2 cell and mouse livers following treatment with tLNP-formulated siNC (chemically modified) (tLNP/siNC) or tLNP/siHBV. As shown in Fig. 4d, e, tLNP/siHBV demonstrated limited sequence-specific off-target effects, interruption on liver transcriptome, and induction of immune responses.

To further facilitate the clinical utilization of siRNA therapeutics, the biosafety profile of tLNP/siHBV was analyzed in mice. A single dose of tLNP/siHBV was intravenously injected at doses of 1 mg/kg and 2.5 mg/kg, respectively. tLNP/siNC was administrated at a dose of 1 mg/kg. Lipopolysaccharide (LPS) and polyI:C were intraperitoneally injected at doses of 5 and 10 mg/kg, and used as positive controls. Blood was sampled at 3, 24, and 48 h. As shown in Fig. 4f–i, LPS induced a remarkable elevation of IL-6, IFN-γ and TNF-α as expected, whereas neither tLNP/siNC nor tLNP/siHBV triggered cytokine expression during 48 h observation period. Intrahepatic inflammatory response-related genes were also determined, confirming the low immunogenicity of tLNP/siHBV (Supplementary Fig. 10).

### tLNP/siHBV exhibits potent anti-HBV activity in rAAV-HBV1.3 and rAAV-rcccDNA/rAAV-Cre mouse models

**Fig. 5:**
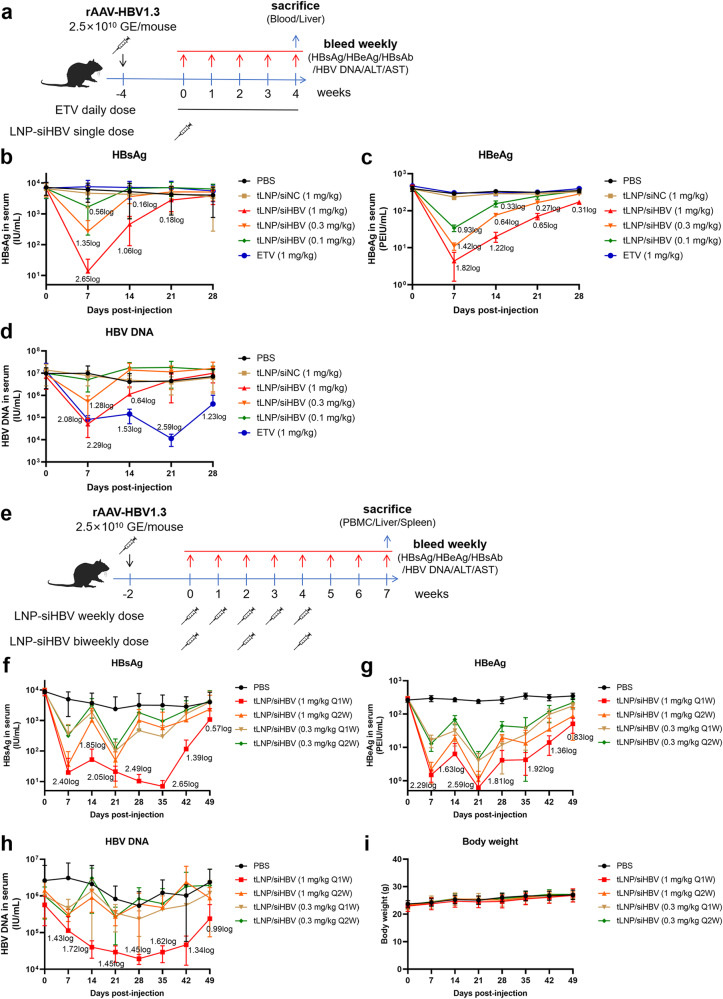
Anti-HBV efficacy of tLNP/siHBV in rAAV-HBV1.3 mouse model. **a** Treatment and sampling schedule of the single-dose study. Mice were injected with rAAV-HBV1.3 (2.5 × 10^10^ viral genome (v.g.) per mice), 2 ~ 4 weeks later, the mice were grouped according to seral HBsAg levels, and treated with PBS, tLNP/siNC (1 mg/kg), tLNP/siHBV of different doses (0.1, 0.3, and 1 mg/kg), or given with Entecavir (ETV, 1 mg/kg) orally. **b** Mice were bled once weekly, and the seral levels of HBsAg, (**c**) HBeAg and (**d**) HBV DNA were monitored during the treatment course. Data were shown as means ± SDs (*n* = 8). **e** Treatment and sampling schedule of the multi-dose study. HBV-replicating mice model was obtained and grouped according to seral HBsAg levels. Mice were then treated with PBS, tLNP/siHBV at the doses of 1 mg/kg and 0.3 mg/kg, and at the frequency of once weekly (Q1W) or once bi-weekly (Q2W). **f** Mice were bled once weekly, and the seral levels of HBsAg, (**g**) HBeAg, and (**h**) HBV DNA during the treatment course were determined by commercial kits. **i** Body weights were monitored during the treatment course. Data were shown as means ± SDs (*n* = 8)

The antiviral efficacy of tLNP/siHBV was further assessed in the rAAV-HBV1.3 mouse model, in which HBV replication was based on an adenovirus-associated virus (AAV) containing a transgene encompassing a 1.3× overlength HBV genome of genotype D. Single doses of tLNP/siHBV at 0.1, 0.3, and 1 mg/kg were intravenously injected to characterize the dose-response and kinetics of the viral products. Negative controls included PBS and tLNP/siNC, while Entecavir (ETV), the first-line antiviral medication for CHB treatment, served as a positive control. Blood was sampled weekly as shown in Fig. 5a and the results suggested that tLNP/siHBV significantly reduced HBsAg (0.56–2.65log_10_ reduction, vs PBS), HBeAg (0.93–1.82log_10_ reduction) and circulating HBV DNA (1.28–2.29log_10_ reduction) in a dose-dependent manner (Fig. 5b–d). In particular, a single dose of tLNP/siHBV (1 mg/kg) achieved 2.65log_10_ IU/mL reduction of HBsAg relative to the PBS control. The reduction in viral products was durable, with resolution of the effects of single-dose treatment occurring between day 7 and 28 in a dose- and time-dependent manner. In contrast, although ETV reduced viral replication with high efficiency, it failed to decrease viral antigen levels throughout the treatment course. Taken together, tLNP/siHBV could not only reduce viral antigen burden, but also block the synthesis of viral DNA.

Repeated doses (two groups, respective 0.3 and 1 mg/kg) of tLNP/siHBV were intravenously injected into mice of HBV replication weekly (five doses in total) or bi-weekly (three doses in total). Blood was sampled weekly for serological testing and observation period continued for three weeks after treatment discontinuation (Fig. 5e). The results showed a significant reduction in HBsAg in a dose- and time-dependent manner (Fig. 5f). Over 2log_10_ IU/mL reduction of viral surface antigen was observed on day 7 after administration, and the inhibition efficiency maintained at 1.85-2.65log_10_ IU/mL (relative to the PBS group) when the dose was 1 mg/kg during the weekly dosing course. Similar effects were seen for HBeAg (1.63–2.59log_10_ reduction) and HBV DNA (1.43–1.72lgo_10_ reduction) levels (Fig. 5g, h). Three weeks after the final dose, 0.57log_10_ IU/mL repression efficiency of HBsAg was still observed. Whereas intrahepatic viral products except HBsAg recovered largely after three weeks of drug withdrawal, which might be due to the stable existence of cccDNA and AAV episome (Supplementary Fig. 11). Toxicity evaluation was performed. Neither body weight changes nor histological abnormalities were observed (Fig. 5i and Supplementary Fig. 12a). Seral anti-HBs antibody levels were determined, and the results showed that one out of eight (1/8, 12.5%) mice produced anti-HBs antibodies after receiving five weekly doses of tLNP/siHBV at doses of 0.3 and 1 mg/kg.

The efficacy of tLNP/siHBV was further evaluated in a rAAV-rcccDNA/rAAV-Cre mouse model, which was established independently to support the long-lasting maintenance of recombinant cccDNA and antigenemia.^47^ The results demonstrated that a single dose of tLNP/siHBV at doses of 0.1, 03, and 1 mg/kg led to a significant reduction of HBsAg levels (1.83-2.74log_10_ IU/mL, relative to the PBS group), and multi-doses of tLNP/siHBV at a dose of 1 mg/kg resulted in a durable reduction in HBsAg levels during treatment courses (Supplementary Fig. 13).

### Combined siHBV and mIL-2 mRNA therapy leads to synergistic viral and immune control of hepatitis B

**Fig. 6:**
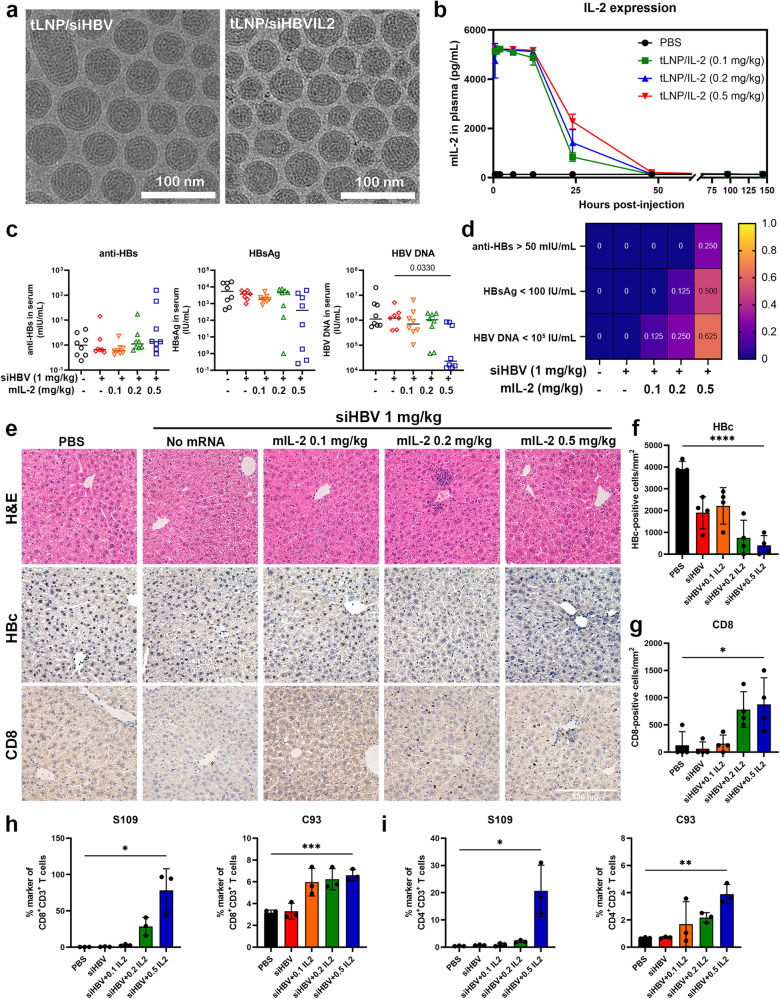
Additive antiviral efficacy of tLNP/siHBVIL2 in rAAV-HBV1.3 mouse model. **a** The morphology of tLNP/siHBV and tLNP/siHBVIL2 was observed by cryogenic electron microscopy (Cryo-EM). **b** mIL-2 mRNA encapsulating tLNP (tLNP/IL2) was intravenously injected into C57BL/6 mice at the doses of 0.1, 0.2, and 0.5 mg/kg, plasma was collected at indicated time points and the mIL-2 expression levels were determined via commercial ELISA kits. Data were shown as means ± SDs (*n* = 3). **c** C57BL/6 mice of HBV replicating models were divided into groups according to seral HBsAg levels, mice were then intravenously injected with PBS, tLNP/siHBV (1 mg/kg) and tLNP/siHBVIL2 (siHBV kept at the dose of 1 mg/kg, and mIL-2 mRNA was given at the doses of 0.1, 0.2, and 0.5 mg/kg). Mice were treated at weekly dose frequency for five doses, then the treatment stopped for another three weeks, the seral viral antigens and DNA were monitored at weekly frequency. The dot plots of anti-HBs, HBsAg and HBV DNA levels at the end of the experiment were displayed with median levels indicated (*n* = 7 ~ 8). **d** Heatmap displaying the percentage of mice of different groups. **e** Histological analysis of liver sections of HBV-replicating mice treated with or without tLNP/siHBV (1 mg/kg) and tLNP/siHBVIL2 (siHBV: 1 mg/kg, mIL-2 mRNA: 0.1–0.5 mg/kg). Immunohistochemical staining of HBc and CD8 levels in liver sections of multi-dose treated mice of HBV replication. Scale bar indicated 100 μm. **f** The HBc- positive cells per mm^2^ liver sections and (**g**) the infiltration of CD8- positive cells per mm^2^ liver sections were displayed. Data were analyzed using unpaired two-tailed Students’ *t* test analysis, and presented as means ± SDs (*n* = 4). **h** The statistical analysis of the proportion of HBsAg- (S109) and core- (C93) specific CD8^+^ and (**i**) CD4^+^ T cells in gated CD3^+^ T cells. Data were analyzed using unpaired two-tailed Students’ *t* test analysis, and presented as means ± SDs (*n* = 3). *P < 0.05, **P < 0.01, ***P < 0.001, and ****P < 0.0001

The desired treatment endpoint is to achieve seroclearance of HBsAg, with or without seroconversion, which is associated with a prolonged prognosis. The siHBV therapy only result in viral control in a limited number of subjects, and immunological evaluation showed that siHBV alone did not alter the phenotype or improve the function of CD8^+^ T cells in the treated mice (Supplementary Fig. 12b–d). We then investigated whether combining siHBV with immunomodulators such as IL-2 could improve therapeutic outcomes. Thus, an mIL-2 encoding mRNA was constructed (see Supplementary Methods), encapsulated within tLNP (tLNP/IL2), and confirmed for proper expression and induction of downstream STAT5 phosphorylation (Fig. 6b and Supplementary Figs. 14 and 15a, b). Intravenous injection of tLNP/IL2 into mice increased the percentage of CD69-positive and CD107a-positive immune cells in the liver, indicating proper expression and functionality (Supplementary Fig. 15c, d). The safety of IL-2 overexpression after tLNP/IL2 injection was also confirmed that then (Supplementary Fig. 15e–h).

The mIL-2 mRNA and siHBV were further encapsulated within a single tLNP (tLNP/siHBVIL2) at weight ratios of 1:10, 1:5, and 1:2, and showed satisfactory size, PDI and ζ potentials, with an encapsulation efficiency of ~97% (Fig. 6a and Supplementary Fig. 16a). The nanoparticles were administrated in five doses in HBV-replicating mouse models at a once weekly dose frequency and continued observation for three weeks after the final dose (Supplementary Fig. 16b). The results showed that tLNP/siHBVIL2 demonstrated potent viral antigen inhibitory efficiency comparable to that of tLNP/siHBV monotherapy, but exhibited higher inhibitory effects on HBV DNA (Supplementary Fig. 16c–f). Specifically, four out of eight (4/8, 50%) mice retained significant HBsAg control (<100 IU/mL) and five out of eight (5/8, 62.5%) retained strong HBV DNA reduction (>2log_10_ reduction) at the end of the observation, two of the mice (2/8, 25%) also produced anti-HBs antibodies (Fig. 6c, d). Furthermore, the proportion of HBc-positive cells in liver sections was significantly reduced (Fig. 6e, f). Other intrahepatic parameters were determined, revealing that tLNP/siHBVIL2 induced more persistent viral replication and transcription inhibition compared to tLNP/siHBV monotherapy, whereas intrahepatic cccDNA levels remained unchanged (Supplementary Fig. 17b–h). Mechanistically, tLNP/siHBVIL2 treatment induced a dramatic increase in the proportion of HBsAg-specific (S109 tetramer-stained) and core-specific (C93 tetramer-stained) CD8^+^ and CD4^+^ T cells in the liver, as observed by flow cytometry (Fig. 6h, i and Supplementary Fig. 17a). Histological studies further indicated that tLNP/siHBVIL2 induced significant infiltration of CD8^+^ T cells into the liver, enabling prolonged viral control without apparent liver injury (Fig. 6e, g and Supplementary Figs. 18 and 19).

## Discussion

Chronic hepatitis B represents a major health burden. Excessive viral burden, especially HBsAg, can mediate immune tolerance and excessive consumption of T cells, which can further lead to viral persistence and immune dysfunction.^48^ RNAi therapeutics target and degrade HBV transcripts with high efficiency, inhibit the HBV antigen expression, block viral replication, and alleviate immune tolerance. Therefore, it is expected to achieve “functional cure” in clinic practices.

For a complex viral genome with high mutations and multiple genotypes, optimal designation of siRNA targeting a conservative sequence is important not only for avoiding potential resistance to RNAi but also for the treatment of multiple genotypes of HBV infection. ARC-520, a first-in-class RNAi for CHB, failed to target the intHBV-derived HBsAg transcripts because the trigger target sites were located in the DR1/DR2 region, which is commonly deleted during integration.^18^ The current generation of RNAi compounds, such as JNJ-3989 and VIR-2218, targets not only cccDNA-derived transcripts, but also those derived from HBV integrates.^49,50^ However, these therapeutics also possess unsatisfactory efficacy due to the delivery system and liver toxicity derived from miRNA-like off-target effects and siRNA-triggered innate immune responses.^27,46,51^ RNAi agents, such as RG6346 and ALG-125755, target HBsAg but not HBx transcripts, whereas HBx is considered an important cccDNA transcriptional regulator.^52,53^ In the present study, an siRNA combination (siHBV) was selected to cover pan-genotypic sequences in the HBV genome (98.55% coverage), which targeted all cccDNA- and intHBV-derived transcripts and possessed limited off-target effects and tissue toxicity. The optimized chemical modification further enhanced serum stability and preserved the knockdown activity of the chosen siHBV.

Furthermore, a modified ionizable lipid nanoparticle based on 3% HO-PEG_2000_-DMG (tLNP), which exhibited excellent siRNA delivery efficiency and biosafety profiles, was developed to overcome the delivery obstacles of siHBV. Mechanistically, optimization of the proportion (from 1.5% to 3%) and terminal group of external PEGylation (using OH-PEG_2000_-DMG instead of mPEG_2000_-DMG) aims to reduce recognition by pre-existing anti-PEG antibodies, improve stability and reduce immunogenicity, avoid off-target uptake by immune cells, prolong circulation time, and improve liver-targeted delivery and efficacy. The delivery performance of the formulation was evaluated using siApoB as a model siRNA. We found that tLNP/siApoB exhibited excellent delivery and knockdown efficacy, with an ED_50_ value of 0.02303 mg/kg, comparable to that of the 1.5% mPEG_2000_-DMG-based LNP formulation (the classic prescription of siRNA-LNP in Onpattro or mRNA-LNP in Spikevax).

The therapeutic efficacy of tLNP/siHBV was evaluated in mouse models of HBV replication. Higher than 2log_10_ IU/mL decrease of viral antigen (2.65log_10_ reduction of HBsAg, 1.82log_10_ reduction of HBeAg) and HBV DNA (2.29log_10_ reduction) were observed after a single dose of tLNP/siHBV at the dose of 1 mg/kg, comparing to that of PBS group. Repeated dosing at the dose of 1 mg/kg and at once weekly dosing frequency could achieve sustained reduction in HBsAg (1.85-2.65log_10_ reduction, vs PBS), HBeAg (1.63-2.59log_10_ reduction) and viral replicates (1.43-1.72log_10_ reduction) during treatment courses, with one in eight (1/8, 12.5%) of mice produced anti-HBs antibody. In addition, toxicity evaluation revealed that tLNP/siHBV was well tolerated by animals at a single dose of 2.5 mg/kg and after multiple doses (five doses in total) at a dose of 1 mg/kg.

Activation of the immune system is the ultimate goal in gaining immune control over persistent HBV infection. Partial immune restoration is anticipated after HBsAg reduction by siRNA, which may provide a “window” to break immune tolerance, thus offering opportunity to construct antiviral immunity within finite therapeutic duration and increase the “functional cure” rate of CHB before arising potential side effects or resistance.^29,30^ However, our studies showed that viral antigen levels restored gradually after the treatment cessation, and few anti-HBs antibodies were detected during the treatment procedures. Meanwhile, tLNP/siHBV treatment did not improve the frequency, phenotype, or function of immune cells in treated mice, indicating that combining siHBV with an immunomodulating agent is warranted. Recent studies have indicated that IL-2 may function as an important cytokine that promotes virus-specific T cell differentiation and proliferation into IFN-γ-producing cytotoxic effector cells.^32–35^ IL-2-based immunotherapy is proposed to revert CD8^+^ T cell dysfunction induced by hepatocellular priming.^54^ Sequential IL-2 administration after receiving PEGIFNα therapy increased the frequency and function of HBV-specific CD8^+^ T cells thus improving clinical outcomes.^36^ Here, our investigation of tLNP/siHBVIL2, which combines siHBV and mIL-2 mRNA, showed sustained HBV inhibition and improved immune control. Specifically, tLNP/siHBVIL2 can provide additive antigenic and immune control of the virus, with four out of eight (4/8, 50%) mice retaining longer HBsAg control (<100 IU/mL), two of which (2/8, 25%) produced anti-HBs antibodies, and five out of eight (5/8,62.5%) mice retained significant HBV DNA reduction (>2log_10_ reduction) at the end of observation. Mechanistically, tLNP/siHBVIL2 inhibited viral burden through siHBV-based RNAi, and triggered the proliferation and infiltration of HBV-specific CD8^+^ and CD4^+^ T cells via expressed mIL-2 cytokines. The co-delivery of siHBV and mIL-2 mRNA utilizing the feasibility of tLNP not only achieved local expression of IL-2 in the liver, prolonged the cytokine half-life, but also enabled innovative combinational therapeutics with a single agent. These findings may assist in the design of future clinical trial aiming to achieve “functional cure” of CHB.

This study has several limitations. First, the clinical stage and medication status of patients may influence the treatment outcomes of RNAi therapeutics. Secondly, although the present siHBV can theoretically target both cccDNA- and intHBV-derived HBsAg transcripts, careful examination of pre-clinical models such as HBV transgenic mice that mimics HBV natural integration, integrative lentivirus-based animal models, or humanized mouse models that permits HBV infection is necessary. Considering that the siRNA trigger against HBx may lost its target sequence after HBV integration, changing the proportions of siRNA triggers against HBs and HBx according to clinical virus parameters of patients may help improve therapeutic outcome. Next, while the rAAV-HBV1.3 model is the most studied model of chronic HBV infection for investigating the effects of anti-HBV therapeutics, it still has limitations.^39^ Unlike nature infections, HBV virus originates from the rAAV-HBV1.3 transcription template rather than the episomal cccDNA template, and is unable to spread. The duration of antigen exposure differs from that in patients who have been infected for decades, with more profound immune tolerance. Besides, considering the potential drawbacks of the different pharmacokinetics of co-delivered siHBV and mIL-2 mRNA, additional optimization of mRNA for enhanced translation and prolonged expression duration, or the creation of a fusion gene combining IL-2 with serum albumin (HSA) or liver-targeting apolipoprotein (ApoA-I) may assist in improving therapeutic outcome. Finally, combinational therapy involving RNAi and an immunomodulatory agent such as therapeutic vaccines and interferon-α has shown promise in clinical trials, and is considered as prospective strategy in the future. More efforts are needed to determine more reasonable combinational strategies, potential synergistic effects, and the underlying mechanisms, which might constitute valuable hints for achieving better clinical outcomes.

In conclusion, the present study provided a promising strategy to achieve sustained and profound reduction in HBsAg and other viral components with high safety by screening pan-genotypic and multifunctional siRNA triggers and optimizing highly efficient and secure tLNP-based delivery system. Further studies using the combined siHBV and mIL-2 mRNA therapeutics with tLNP as nucleic acid carriers, may have the potential to become a cornerstone therapeutic approach for treating CHB.

### Supplementary information

Supplementary Materials

Supplementary Materials

## Data Availability

Data are available on reasonable request. Most data relevant to the study are included in the article or uploaded as supplementary information. Additional data are available upon reasonable request.
