# Supplementary material for: Optimized RNA interference therapeutics combined with interleukin-2 mRNA for treating hepatitis B virus infection
Source: Signal Transduct Target Ther. 2024 Jun 21;9:150. doi: 10.1038/s41392-024-01871-8 (PMC11189933; doi:10.1038/s41392-024-01871-8)
Supplement: Supplementary file 1 — Supplementary Materials [file 41392_2024_1871_MOESM1_ESM.docx]

Supplementary Materials for

Optimized RNA Interference Therapeutics Combined with Interleukin-2 mRNA for Treating Hepatitis B Virus Infection

Wenjing Zai^1, #^, Min Yang^2, 4, #^, Kuan Jiang^2,6^, Juan Guan^7^, Huijing Wang^5^, Kongying Hu^1^, Chao Huang^1^, Jieliang Chen^1^, Wei Fu^5, *^, Changyou Zhan^2, 4, *^, Zhenghong Yuan^1, 3, *^

Correspondence to: [zhyuan@shmu.edu.cn](mailto:zhyuan@shmu.edu.cn); [cyzhan@fudan.edu.cn](mailto:cyzhan@fudan.edu.cn); [fuweizhulu@163.com](mailto:fuweizhulu@163.com)

^#^ These authors contributed equally to this work.

1 Key Laboratory of Medical Molecular Virology (MOE/NHC/CAMS), Research Unit of Cure of Chronic Hepatitis B Virus Infection (CAMS), Shanghai Frontiers Science Center of Pathogenic Microbes and Infection, School of Basic Medical Sciences, Shanghai Medical College, Fudan University, Shanghai, 200032 P. R. China.

2 Department of Pharmacology, School of Basic Medical Sciences, Fudan University, Shanghai, 200032 P. R. China.

3 Shanghai Institute of Infectious Disease and Biosecurity, Shanghai, 200032 P. R. China.

4 Shanghai Engineering Research Center for Synthetic Immunology, Fudan University, Shanghai, 200032 P. R. China.

5 Institute of Pediatric Translational Medicine, Shanghai Institute for Pediatric Congenital Heart Disease, Shanghai Children's Medical Center, School of Medicine, Shanghai Jiao Tong University, Shanghai 200127, China.

6 Eye Institute and Department of Ophthamology, Eye and ENT Hospital, Fudan University, Shanghai 200030 P. R. China.

7 Pharmacy Department of Huashan Hospital, Fudan University, Shanghai 200040 P. R. China.

* Correspondence to:

Professor Zhenghong Yuan, Key Laboratory of Medical Molecular Virology (MOE/NHC/CAMS), School of Basic Medical Sciences, Shanghai Medical College, Fudan University, Shanghai, China; [zhyuan@shmu.edu.cn](mailto:zhyuan@shmu.edu.cn)

Professor Changyou Zhan, Department of Pharmacology, School of Basic Medical Sciences, Fudan University, Shanghai, China; [cyzhan@fudan.edu.cn](mailto:cyzhan@fudan.edu.cn)

Professor Wei Fu, Institute of Pediatric Translational Medicine, Shanghai Institute for Pediatric Congenital Heart Disease, Shanghai Children's Medical Center, School of Medicine, Shanghai Jiao Tong University, Shanghai, China; [fuweizhulu@163.com](mailto:fuweizhulu@163.com)

**This PDF file includes:**

Materials and Methods

Figures. S1 to S19

Tables S1 to S5

Materials and Methods

Preparation and characterization of tLNP

The octanoicacid,8-[(2-hydroxyethyl)[6-oxo-6-(undecyloxy)hexyl]amino]-,1-octylnonylester (SM-102), 1,2-Distearoyl-sn-glycero-3-phosphorylcholine (DSPC), 1,2-dimyristoyl-rac-glycero-3-methoxylpolyethylene glycol-2000 (mPE_G2000_-DMG) and cholesterol were purchased from AVT Pharmaceutical Co., Ltd. (Shanghai, China). Additionally, 1,2-dimyristoyl-rac-glycero-3-hydroxylpolyethylene glycol-2000 (HO-PEG_2000_-DMG) was synthesized independently. tLNP was prepared by the process of spontaneously vesicle formation. In brief, SM-102, DSPC, HO-PEG_2000_-DMG, and cholesterol were dissolved in ethanol and mixed to yield a molar ratio of 50:10:3:38.5. The nitrogen phosphorus ratio of tLNP to siRNA is 3 (the nitrogen phosphorus ratio of tLNP to mRNA is 6. siRNA was dissolved in 6.25 mM sodium acetate buffer (pH 5), and then mixed with lipid mixture at approximately 3:1 (v/v) using a microfluidic chip (PDMS 11#, MPE-L1s，Suzhou Aitesen Pharmaceutical Equipment Co.,Ltd., China). The total mixing flow rate was 1 mL/min. Preparations were dialyzed with PBS (pH7.2) or 20 mM Tris (pH7.4) containing 8% sucrose in Slide-A-Lyzer dialysis cassettes (Thermo Scientific, Rockford, IL, USA) for at least 15 h. The resulting tLNPs were centrifuged in Amicon ultrafiltration tubes (MWCO 100 kDa, Millipore, Billerica, MA, USA) to remove the remaining organic solvent and free compounds, and 1× PBS was used to concentrate or dilute the solution to desired concentration.

The particle size, surface charge (ζ potential), and polydispersity index (PDI) of lipid nanoparticles were analyzed using Dynamic Light Scattering (DSL) (Malvern Nano ZS, Malvern Panalytical, England). The morphology of LNPs was observed by cryogenic electron microscopy (Cryo-EM) (FEI Tecnai G2 F20 TEM) operating at 200 kV.

Encapsulation efficiency

The encapsulation efficiency of LNPs was analyzed by RiboGreen RNA assay (Invitrogen, Carlsbad, CA).^1^ A standard curve was prepared by measuring the RiboGreen fluorescence value in samples with known concentrations of siRNA/mRNA used in each preparation. To determine siRNA or mRNA entrapment, the signal of the RNA binding dye RiboGreen was compared between formulation samples in the absence and presence of the detergent 2% Triton X-100. The signal comes only from unentrapped RNA in the absence of detergent, whereas the signal comes from total RNA in the presence of detergent. The percentage of encapsulated RNA was measured as follows:

RNA encapsulation efficiency (%) = (concentration of total RNA - concentration of unentrapped RNA) / (concentration of total RNA) × 100%

Serum stability

To improve the serum stability and to reduce immunogenicity, the siRNAs were partially modified at desired sites, or fully chemically modified with alternating 2'-O-methyl group (2'-OMe), 2'-Fluro (2'-F), and the sulfate bond modification (PS) in the backbone, or modified with advanced enhanced stabilization chemistry (aESC) with or without liver-targeting N-acetylgalactosamine (GalNAc) conjugates. The naked unmodified and modified siRNAs were incubated with 10% FBS at 37 ℃. Aliquots were taken at 0, 1, 2, 4, 6, 8, 24 and 48 h, and immediately frozen at -80 ℃ post-incubation. Samples were then mixed with loading buffer, and then subjected to 2% agarose gel electrophoresis and visualized by gel-red staining.

Cy5-siRNA uptake analysis

Cy5-labeled siRNA was purchased from RiboBio (Guangzhou, China), encapsulated within LNP, and intravenously injected into mice at the doses of 0.5 and 0.05 mg/kg. Four hours post-treatment, mice were sacrificed, hepatocytes and liver-associated lymphocytes were isolated by perfusion and collagen IV digestion then purified by density gradient centrifugation. The liver-associated lymphocytes were applied for cell surface staining using anti-mouse CD3 (Brilliant Violet 605^TM^, BioLegend), anti-mouse F4/80 (APC/Cyanine7, BioLegend) and anti-mouse CD19 (Alexa Fluor® 700, BioLegend) antibodies. Data were collected using Attune NxT flow cytometer (Thermo Fisher, USA) and analyzed using FlowJo^TM^ Version 10 software (Tree Star, Ashland, OR). The percentage of Cy5-positive cells in gated cells or mean fluorescence intensity of Cy5 were displayed.

The splenocytes were obtained by passing spleens through 70-μm cell strainers, centrifuged, followed by red blood cell lysis, washed with PBS, and suspended with FACS buffer for subsequence analysis.

*In vivo* biodistribution of LNPs/Cy5-siRNA

C57BL/6 mice were injected with Cy5-siRNA-encapsulating LNPs (at the dose of 0.5 mg/kg), different organs (lung, heart, liver, spleen, and kidney) of mice were collected at 4 hours post-injection, and the intra-organ Cy5 fluorescence was visualized by live imaging (IVIS^®^ Lumina III, PerkinElmer, USA). Mean fluorescence intensity was calculated and presented. Tissues were homogenized in 5% Triton X-100 in PBS, the protein concentrations were determined via BCA (Beyotime Biotechnology, Nantong, China) analysis, and the Cy5 fluorescence was determined by a fluorescence reader.

ApoB knockdown in normal C57BL/6 mice

siRNA targeting against apolipoprotein (ApoB) (siApoB, sense strand: 5’-GUCAUCACACUGAAUACCAAUdTdT-3’, anti-sense strand: 5’-AUUGGUAUUCAGUGUGAUGACdTdT-3’) was utilized to evaluate the delivery efficiency of LNPs. In brief, male C57BL/6 mice were randomly divided into five groups with four mice per group. Then they were administered intravenously with 1× PBS, or varying formulations of siApoB encapsulating LNPs at doses of 0.5 and 0.05 mg/kg, respectively. After 72 h, animals were sacrificed, blood samples and tissue samples were collected. Liver samples were homogenized, followed by total RNA extraction, reverse transcription, and real time quantitative PCR (RT-qPCR) according to the manufacture’s instruction. β-Actin was selected as the reference gene. The triglyceride (TG) and total cholesterol (T-CHO) lipid contents in the liver or in the circulation were extracted and determined via commercial detecting kits according to the manufacture’s protocols (Nanjing Jiancheng Bioengineering Institute, China).

Male C57BL/6 mice were randomly divided into groups with four mice per group. Then they were administered intravenously with 1× PBS, or varying doses of siApoB formulated tLNP, respectively. The doses of tLNP-siApoB were 0.001, 0.01, 0.05, 0.1, 0.5, and 1 mg/kg. After 72 h, animals were sacrificed, blood samples and tissue samples were collected. Liver samples were homogenized, followed by total RNA extraction, reverse transcription, and RT-qPCR quantification according to the manufacture’s instruction. β-Actin was selected as the reference gene. The median effective dose (ED_50_) of the formation was then calculated by GraphPad Prism software. The triglyceride (TG) and total cholesterol (T-CHO) lipid contents in the liver were extracted and detected with commercial detecting kits according to the manufacture’s protocols.

The efficacy of tLNP/siHBV in rAAV-rcccDNA/rAAV-Cre mouse model

The rAAV-rcccDNA/rAAV-Cre mouse model was constructed in-house and was modified from the rAAV-rccdDNA/Alb-Cre mouse model as we previous reported.^2^ In brief, male C57BL/6 mice were inoculated with 2.5×10^10^ vector genomes (v.g.)/mice of rAAV-rcccDNA virus carrying a *loxP* sites flanking monomeric linear HBV sequence (GenBank accession no. V01460.1) and equal amount of rAAV-Cre virus which can express Cre recombinase protein under the CMV promoter. This mouse model can establish long-term maintenance of recombinant cccDNA and antigenemia in hepatocytes. Animals were bled one day before start of treatment and divided into groups to obtain similar HBsAg and HBeAg levels. The mice were then treated with single-dose or multidose of tLNP/siHBV at indicated dosages. Blood samples were collected weekly during the experiments and were applied for relative detections.

Toxicity studies

Male C57BL/6 mice were randomly divided into six groups with five mice per group. Then they were treated with formulations as following: (1) PBS; (2) tLNP/siNC (1 mg/kg); (3) tLNP/siHBV (1 mg/kg); (4) tLNP/siHBV (2.5 mg/kg); (5) lipopolysaccharides (LPS) (5 mg/kg); (6) poly I:C (10 mg/kg). LPS and poly I:C were administrated by intraperitoneal (i.p.) injection. Blood samples were collected at 3, 24 and 48 h after injection. Serum cytokine levels including IFN-γ, TNF-α and IL-6 were determined by ELISA assays (Absin, China) according to manufactures’ introduction.

As for multi-dose treated mouse samples, seral chemistry levels of alanine transaminase (ALT), aspartate transaminase (AST), creatinine (CRE), uric acid (UA), albumin (ALB), lactate dehydrogenase (LDH), and total bilirubin (TBIL) were measured via commercial kits (Nanjing Jiancheng, China). Mice organs were collected at the end of experiment and fixed in 10% formaldehyde, embedded in paraffin, and then sectioned for H&E staining for histological analysis with an optimal microscope.

Bioinformatics and RNA sequencing

HepG2 cells were incubated with tLNP/siNC or tLNP/siHBV at the dose of 50 nM for 16 h. Mice were intravenously injected with tLNP/siNC or tLNP/siHBV at the dose of 1 mg/kg for 72 h, then liver samples were collected and snap frozen. RNA was extracted by TRIzol and cDNA libraries were prepared for following RNA sequencing, according to manufactures’ instructions. Data were processed as described before.^3^ In brief, the fastq. files were trimmed by Cutadapt v3.1 and then applied to human or mouse reference genome by Hisat2, sorted by SAMtools and analyzed for expression using featureCounts. Differential gene expression analysis was performed using DEseq2 using fold change of ≥ 2 and *P* value of ≤ 0.05 as the cutoff.

Hepatocytes, liver-associated lymphocytes and splenocytes isolation

A two-step isolation procedure was utilized to isolate hepatocytes and liver-associated lymphocytes. In brief, liver of mice was perfused with perfusion buffer via portal vain then digested by perfusing with 0.05% Collagenase IV (Yeasen Institute of Biotechnology, Shanghai, China) at 37 ℃ for 3~5 min. The isolated cells were then purified with 40% Percoll (GE Healthcare Life Sciences, Shanghai, China) to obtain hepatocytes and liver-associated lymphocytes for culture or flow cytometry detection.

Spleen of mice were obtained and minced through a 100 μm cell strainer to make a single-cell suspension. The red blood cells were removed with ammonium-chloride-potassium (ACK) buffer (0.83% NH4Cl/0.1% KHCO3).

prcccDNA+pCMV-Cre mouse model

The prcccDNA+pCMV-Cre mouse models were established according to previous description.^4^ In brief, siNC or siHBV were mixed with prcccDNA+pCMV-Cre plasmid (4 μg each) in a volume of PBS equivalent to 8% of the mouse body weight then intravenously injected into mice within 5 to 8 s. Blood were sampled at 2 days, 4 days and 7 days post-injection, and sera HBV antigen levels were determined via commercial kits.

Stability of tLNP

The tLNP was prepared and tested for particle size PDI, potential, and nucleic acid encapsulation efficiency under different storage temperature conditions (including 4, 25, and -20 degrees Celsius). Measurements were conducted every week for a total of 4 weeks.

mIL-2 mRNA synthesis and examination

mRNA was synthesized *in vitro* using T7 RNA polymerase-mediated transcription from a linearized DNA template, which incorporates the 5’and 3’UTRs and a poly-A tail as previously described.^5^ RNA was purified using Ambion MEGA clear spin columns and then treated with Antarctic Phosphatase (New England Biolabs) for 30 min at 37 ℃ to remove residual 5′-phosphates. Treated RNA was re-purified and quantified by Nanodrop one (Thermo Scientific). After purification, mRNA was resuspended in 10 mM Tris HCl, 1 mM EDTA at 1μg/μl for use. In mRNA, uridine was fully replaced by N1-methylpseudouridine. Open reading frame sequence for mouse IL-2 mRNA is provided as follow.

Open reading frame sequence for construction of mouse IL-2 mRNA：atgtacagcatgcagctcgcatcctgtgtcacattgacacttgtgctccttgtcaacagcgcacccacttcaagctccacttcaagctctacagcggaagcacagcagcagcagcagcagcagcagcagcagcagcagcacctggagcagctgttgatggacctacaggagctcctgagcaggatggagaattacaggaacctgaaactccccaggatgctcaccttcaaattttacttgcccaagcaggccacagaattgaaagatcttcagtgcctagaagatgaacttggacctctgcggcatgttctggatttgactcaaagcaaaagctttcaattggaagatgctgagaatttcatcagcaatatcagagtaactgttgtaaaactaaagggctctgacaacacatttgagtgccaattcgatgatgagtcagcaactgtggtggactttctgaggagatggatagccttctgtcaaagcatcatctcaacaagccctcaatga.

To examine the successful construction of mIL-2 mRNA, the mRNA was encapsulated with tLNP with or without siHBV, then incubated with HEK293T cells or intravenously injected into mice. Then the mIL-2 protein levels in the supernatant or mouse serum were determined by commercial ELISA kits (Absin, China). The supernatant was then incubated with mouse splenocytes (mSplenocytes) and the phosphorylation levels of STAT5 (p-STAT5-Tyr694) (Cell signaling) were determined via western blot analysis. Liver-associated lymphocytes were collected and applied for cell surface staining with anti-mouse CD3, anti-mouse CD4, anti-mouse CD8 (Brilliant Violet™ 711, eBioscience), anti-mouse NK1.1 (Alexa Fluor 700, eBioscience), anti-mouse CD11c (APC, eBioscience), anti-mouse CD69 (PE, eBioscience) and anti-mouse CD107a (eFluor™ 450, eBioscience) antibodies. Dead cells were excluded from analysis by fixable viability dye eF780 (eBioscience, Frankfurt, Germany) staining. The percentage of CD69- and CD107a- positive cells in gated populations were calculated by Flow Jo™ software (version 10).

The antiviral efficacy of tLNP/siHBVIL2 were evaluated in rAAV-HBV1.3 mouse model. In brief, tLNP/siHBV (1 mg/kg) or tLNP/siHBVIL2 of varying doses (siHBV: 1mg/kg, mIL-2 mRNA: 0.1, 0.2 and 0.5 mg/kg) were intravenously injected once weekly (five doses in total), then the mice were left untreated for another three weeks. The serum samples were collected weekly, and were applied for HBsAg, HBeAg and HBV DNA analysis according to the manufacturer’s instructions. The mice were sacrificed at the end of experiment, and blood and tissue samples were collected for further detection.

Statistical analysis

The data were expressed as the means ± standard deviations (SDs) or means ± standard error of means (SEMs). One-way or two-way analysis of variance (ANOVA) was applied for multiple comparisons. Significance was defined as **P*<0.05, ***P*<0.01, ****P*<0.001, and *****P*<0.0001.

Figure. S1.


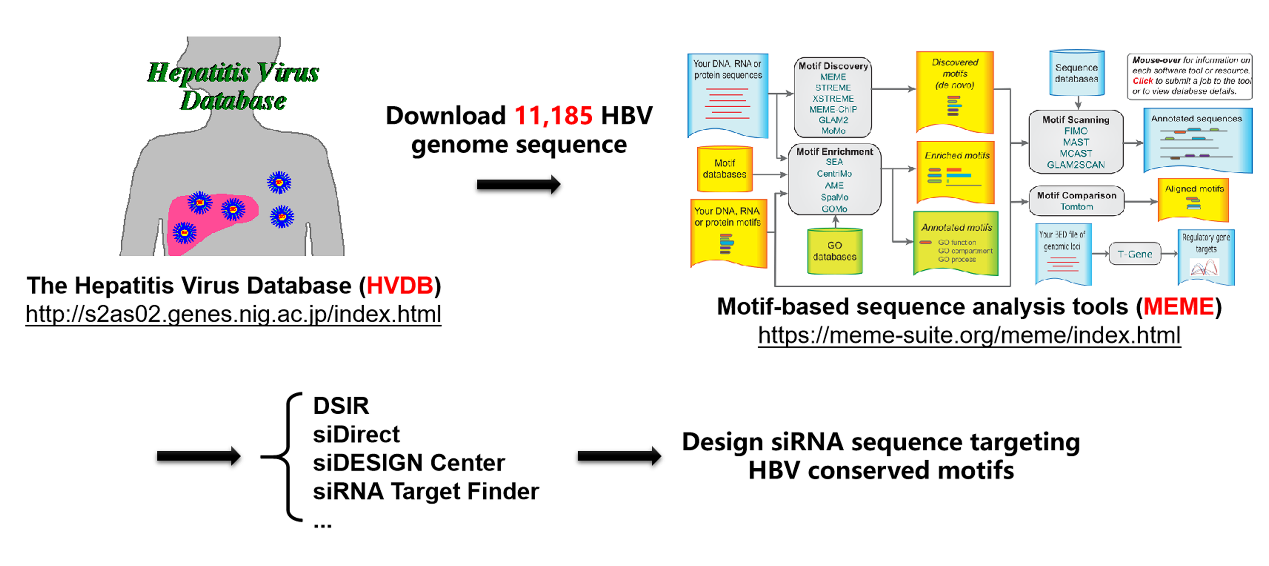


**Supplemental Fig.S1 The designation of effective and conservative anti-HBV siRNA triggers.** Schematics of the designation of effective and conservative anti-HBV siRNA triggers. A collection of 11,185 HBV genome sequences were downloaded from the Hepatitis Virus Database (HVDB) websites, highly conserved motifs were identified via motif-based sequence analysis tools (MEME), then the siRNAs targeting conserved motif sequences of HBV were identified from siRNA designation websites (DSIR, siDirect, siDESIGN Center, siRNA Target Finder, et al.), and were synthesized for further examination.

Figure. S2.


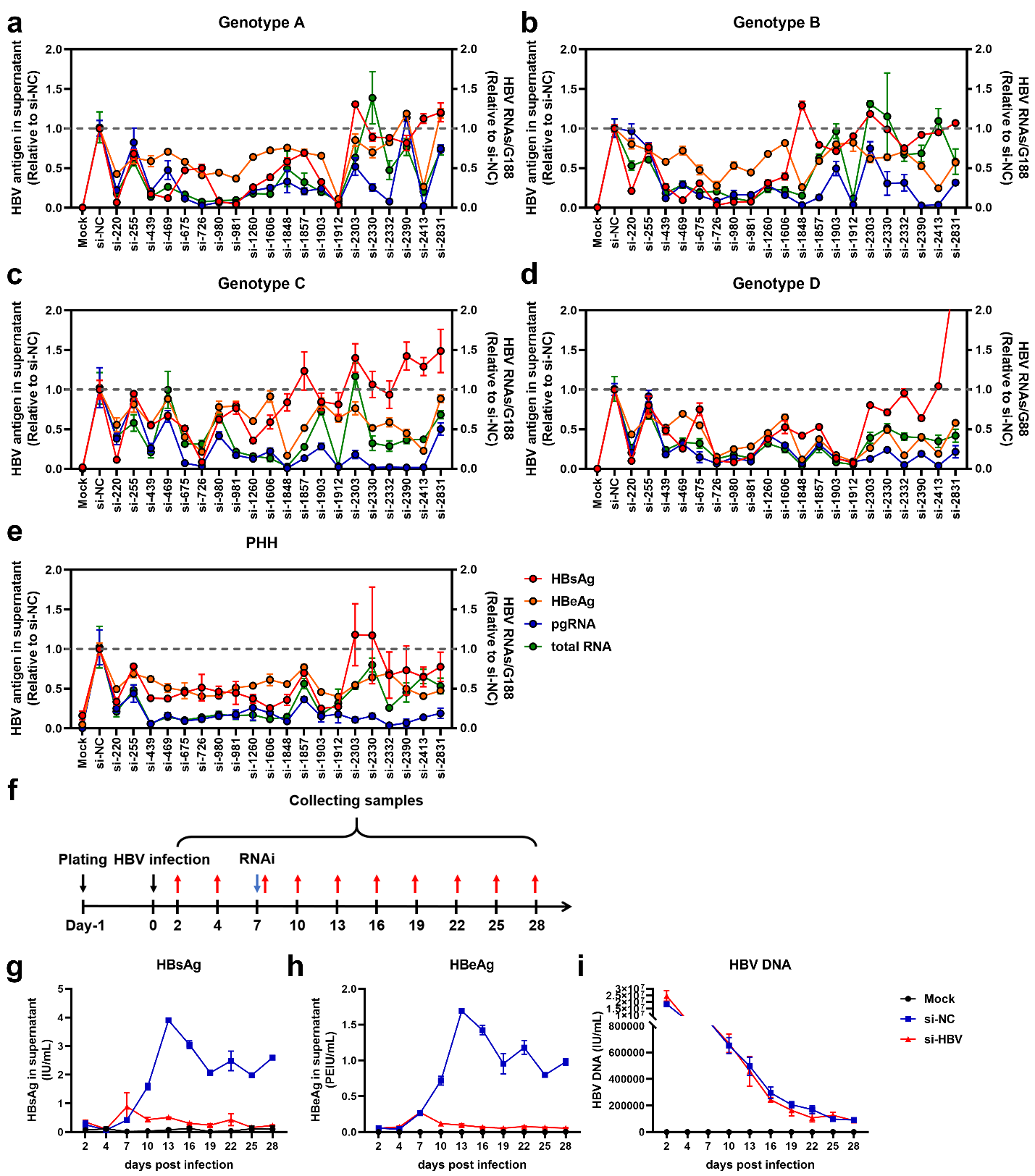


**Supplemental Fig.S2 Screening of siRNAs in different HBV genotypes and cell models.** (a) HepG2 cells were co-transfected with siRNA at the dose of 50 nM and the genotype A, (b) genotype B, (c) genotype C and (d) genotype D of 1.3 × overlength HBV replicon plasmids with Lipofectamine^TM^ 3000 (Invitrogen) transfection reagents, the expression levels of HBsAg and HBeAg in the supernatant were detected at 72 h post-transfection, and the intracellular levels of pgRNA and total RNAs were analyzed via RT-qPCR, comparing to that of control siRNA (si-NC). (e) Five-compound cultured primary human hepatocytes (5C-PHHs) were infected with HBV at a MOI of 200, then transfected with siRNA at the dose of 50 nM with transfection reagents. Expression levels of HBV antigens and intracellular HBV RNAs were then determined at 72 h post-transfection. (f) The treatment schematics were shown. 5C-PHHs were infected with HBV at a MOI of 200, then transfected with siRNA at the dose of 50 nM with transfection reagents at 7 days post-infection. (g) The supernatants were collected at indicated time points and applied for HBsAg, (h) HBeAg, and (i) HBV DNA determination via commercial kits. Data were shown as means ± SDs (n = 3).

Figure. S3.


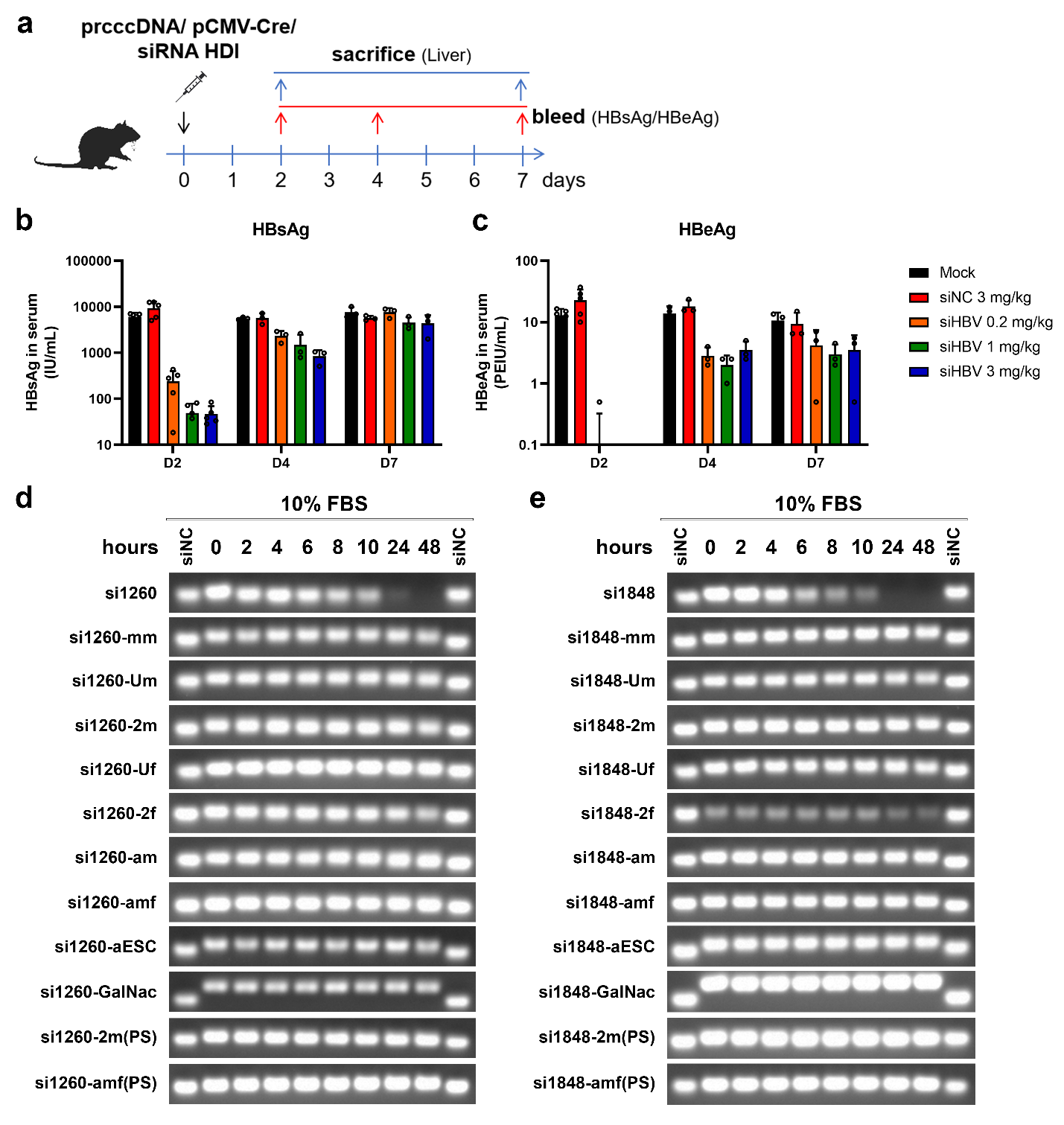


**Supplemental Fig.S3 The anti-HBV efficacy and serum stability of siHBV.** (a) The unmodified dissociative negative control siNC or siHBV were mixed with prcccDNA (4 μg) plus pCMV-Cre (4 μg) plasmids, and then injected into C57BL/6 mice by hydrodynamic injection at varying doses (0.2, 1 and 3 mg/kg). Blood was sampled at indicated time points. (b) The serum HBsAg and (c) HBeAg levels were determined at indicated time points (2, 4 and 7 days). Data were shown as means ± SDs (n = 5). (d) The unmodified and modified si-1260 and (e) si-1848 were incubated with 10% FBS in PBS for indicated durations, and the stability of siRNAs was visualized by gel-red and photographed by imaging system.

Figure. S4.


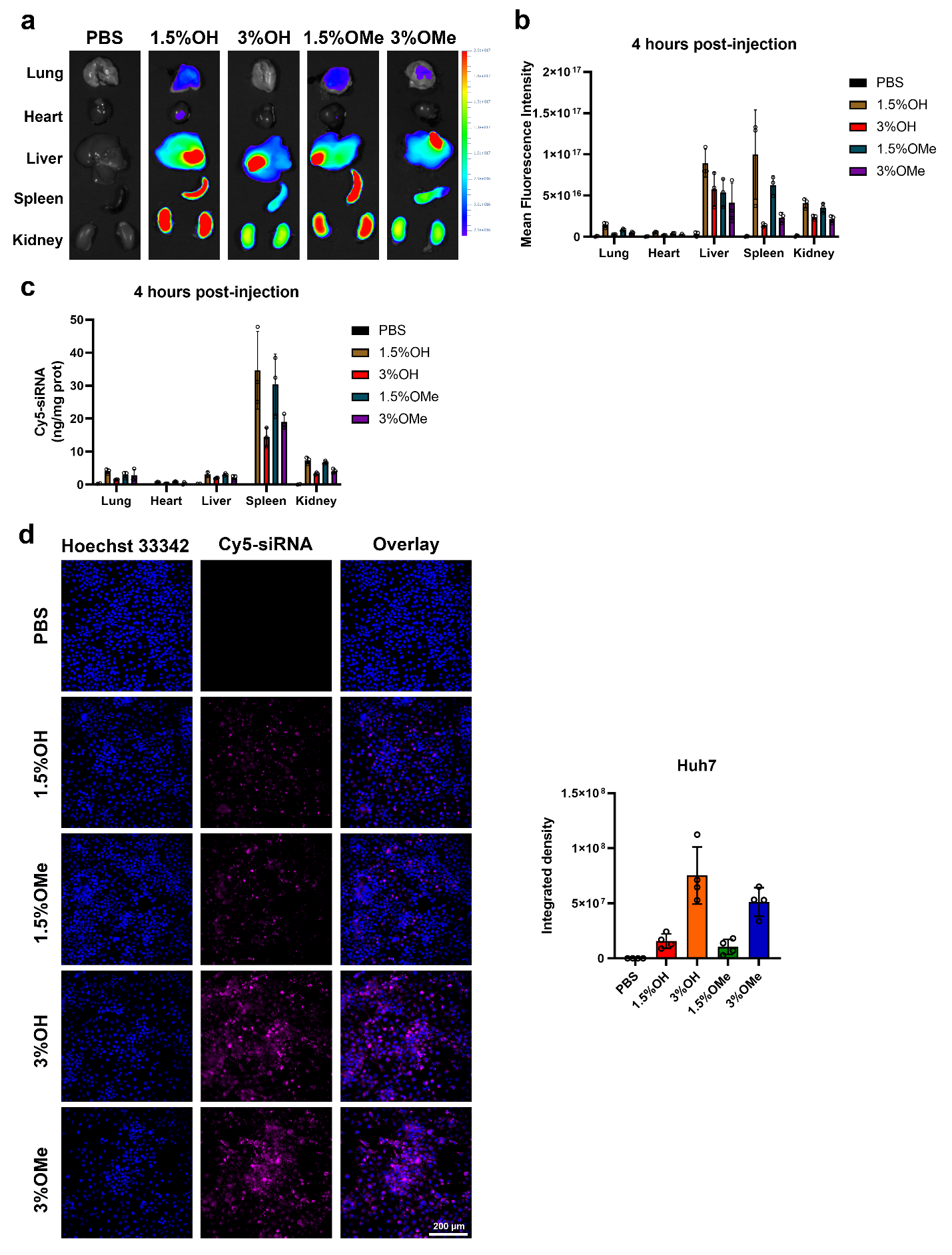


**Supplemental Fig.S4 *In vivo* biodistribution of LNPs of different formula.** (a) C57BL/6 mice were intravenously injected with different formulations of LNPs/Cy5-siRNA at the dose of 0.5 mg/kg, the *in vivo* biodistribution of Cy5-siRNA was visualized via live imaging, and (b) the mean fluorescent intensity was presented as means ± SDs (n = 3). (c) Organs were homogenized in 5% Triton X-100 in PBS, and the fluorescence intensity of the Cy5-siRNA relative to protein concentrations was measured. Data were shown as means ± SDs (n = 3). (d) Huh7 cells were transfected with different formulations of LNPs/Cy5-siRNA at the concentration of 50 nM for 4 h, and the intracellular Cy5-siRNA signaling was visualized via confocal microscopy. Scale bar indicated 200 μm. The integrated intensity of Cy5 signaling was presented as means ± SDs (n = 4).

Figure. S5.


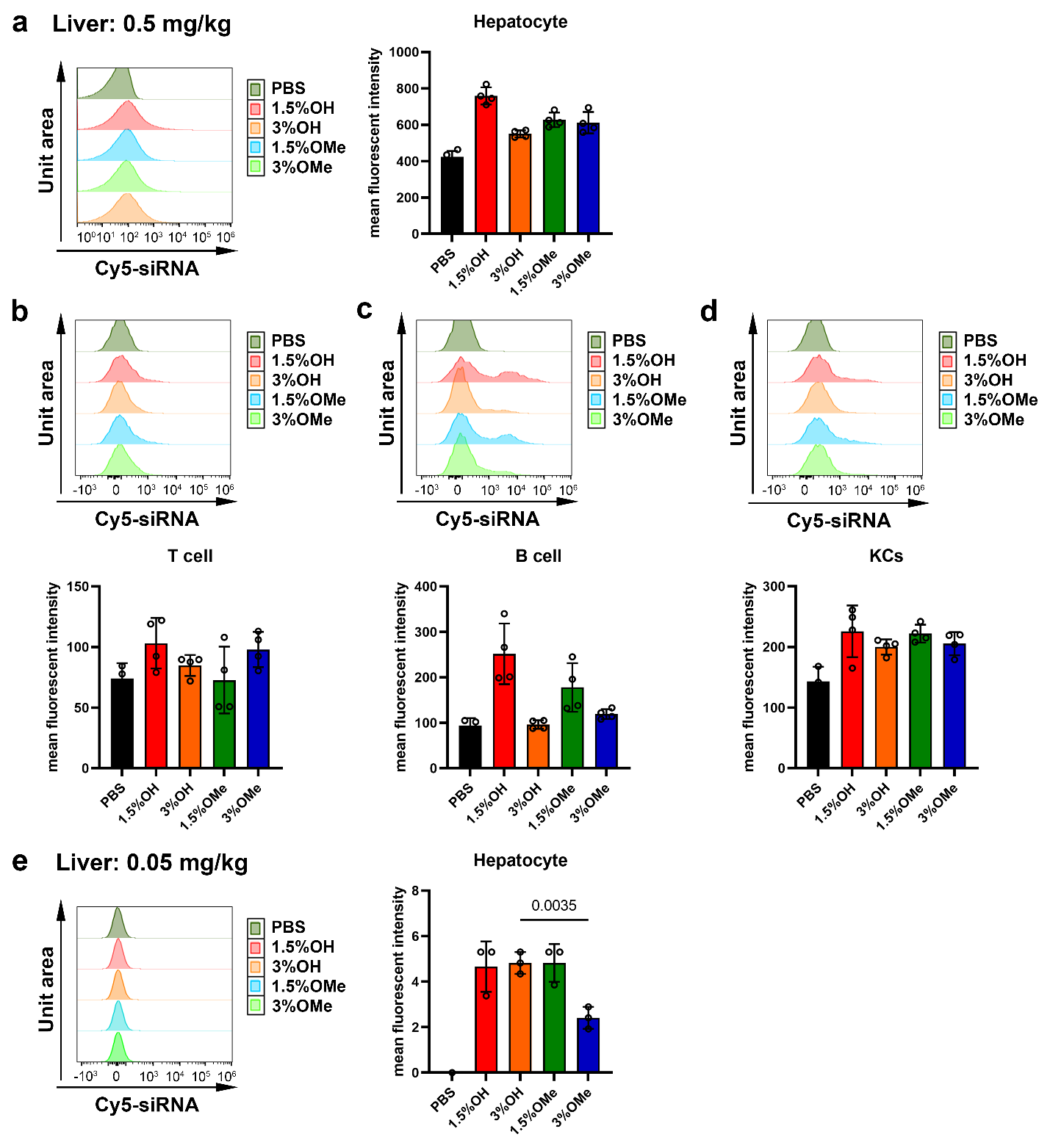


**Supplemental Fig.S5 *In vivo* uptake of LNPs in liver parenchymal and non-parenchymal cells.** (a-d) C57BL/6 mice were intravenously injected with different formulations of LNPs/Cy5-siRNA (at the dose of 0.5 mg/kg), the liver parenchymal and non-parenchymal cells were collected 4 h post-injection, the mean fluorescent intensity of Cy5 in different cells was determined by flow cytometry analysis, and calculated by Flow Jo™ (version 10). Data were presented as means ± SDs (n = 4). (e) The mean fluorescent intensity of Cy5 in hepatocyte when dosed at 0.05 mg/kg was determined. Data were analyzed using unpaired two-way Student’s *t*-test analysis, and presented as means ± SDs (n = 3).

Figure. S6.


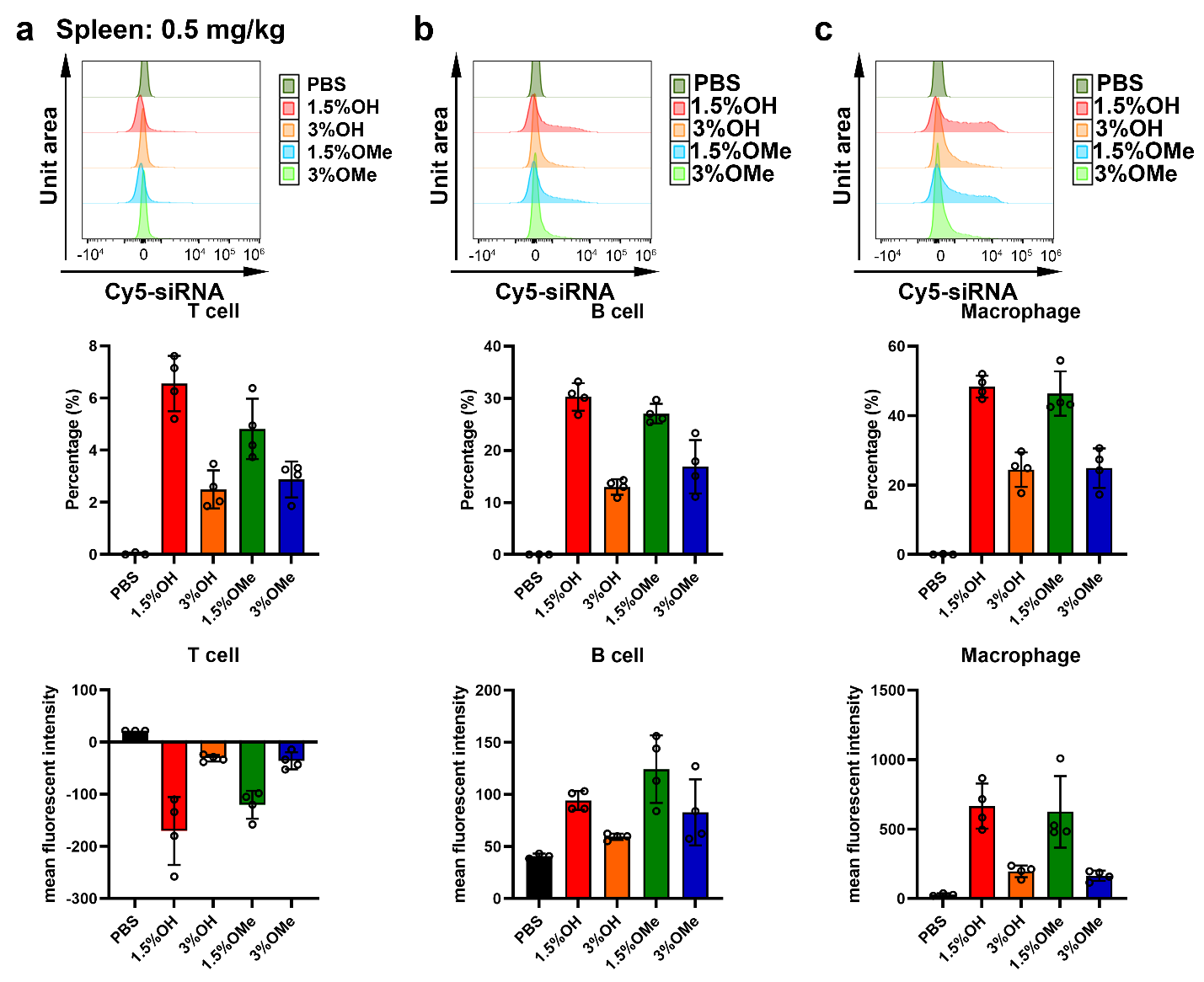


**Supplemental Fig.S6 *In vivo* uptake of LNPs in splenocytes.** (a) C57BL/6 mice were intravenously injected with different formulations of LNPs/Cy5-siRNA (at the dose of 0.5 mg/kg), the splenocytes were collected 4 h post-injection, the mean fluorescent intensity of Cy5 in T cells, (b) B cells, and (c) macrophages was determined by flow cytometry analysis, and calculated by Flow Jo™ (version 10). Data were presented as means ± SDs (n = 4).

Figure. S7.


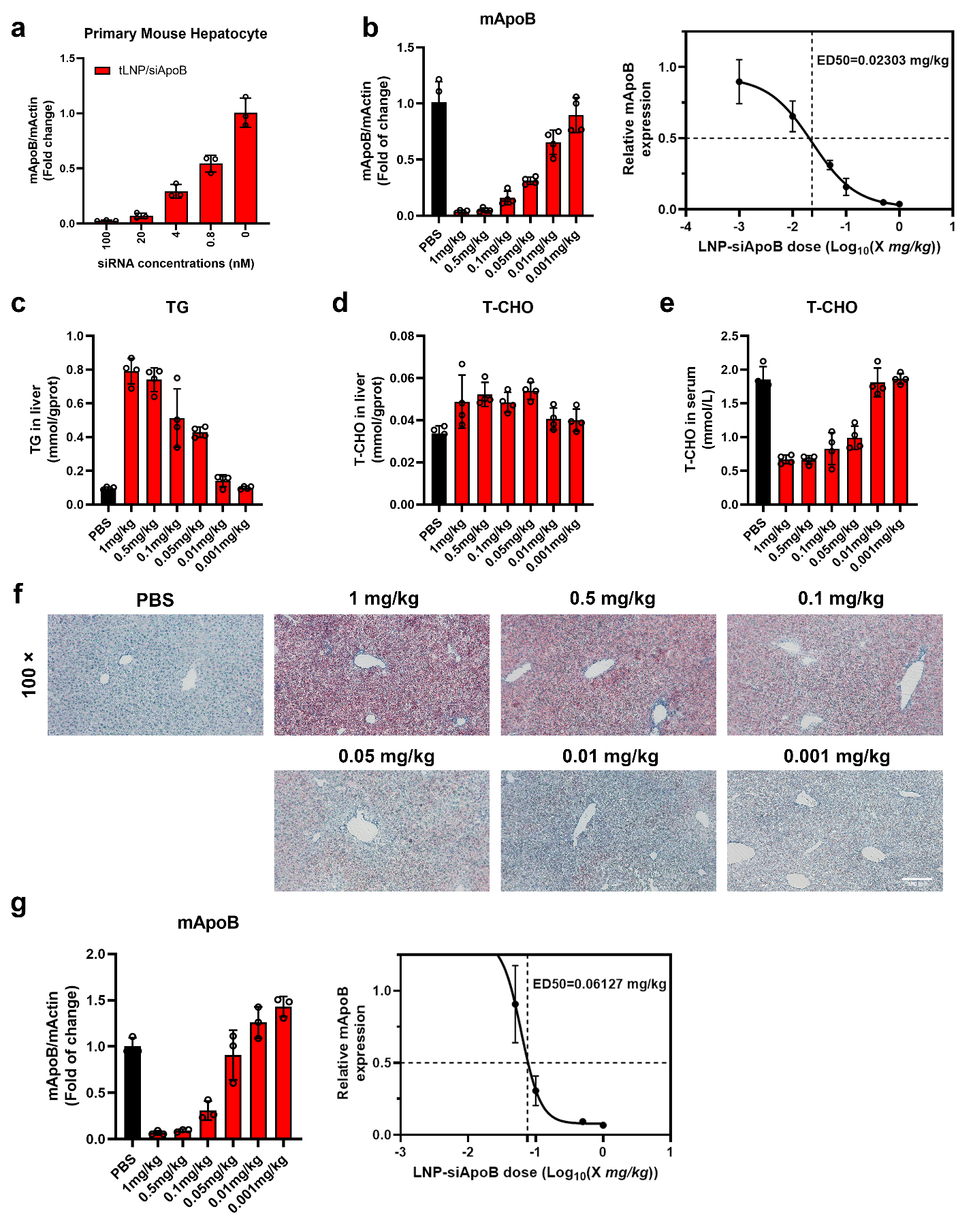


**Supplemental Fig.S7 Liver-targeted delivery efficiency of tLNP.** (a) Primary mouse hepatocytes (PMHs) were treated with different concentrations of tLNP/siApoB (0, 0.8, 4, 20, and 100 nM) for 24 h, then the *ApoB* mRNA levels compared with *β-actin* mRNA levels were determined via RT-qPCR analysis. Data were shown as means ± SDs (n = 3). (b) C57BL/6 mice were intravenously injected with different doses of tLNP/siApoB (0, 0.001, 0.01, 0.05, 0.1, 0.5 and 1 mg/kg), the relative levels of *ApoB* mRNA against *β-actin* mRNA were determined at 72 h post-injection, and the ED_50_ value was calculated by GraphPad Prism software (Version 9.00, San Diego, California, USA). (c) The intrahepatic total triglycerides (TG) and (d) T-CHO levels and (e) seral T-CHO levels were detected via commercial kits. Data were shown as means ± SDs (n = 4). (f) Oil Red O staining of liver sections. Scale bar indicated 100 μm. (g) C57BL/6 mice were intravenously injected with different doses of 3% OMe-PEG_2000_-DMG based LNP-encapsulating siApoB (0, 0.001, 0.01, 0.05, 0.1, 0.5 and 1 mg/kg), the relative levels of *ApoB* mRNA against *β-actin* mRNA were determined at 72 h post-injection, and the ED_50_ value was calculated. Data were shown as means ± SDs (n = 3).

Figure. S8.


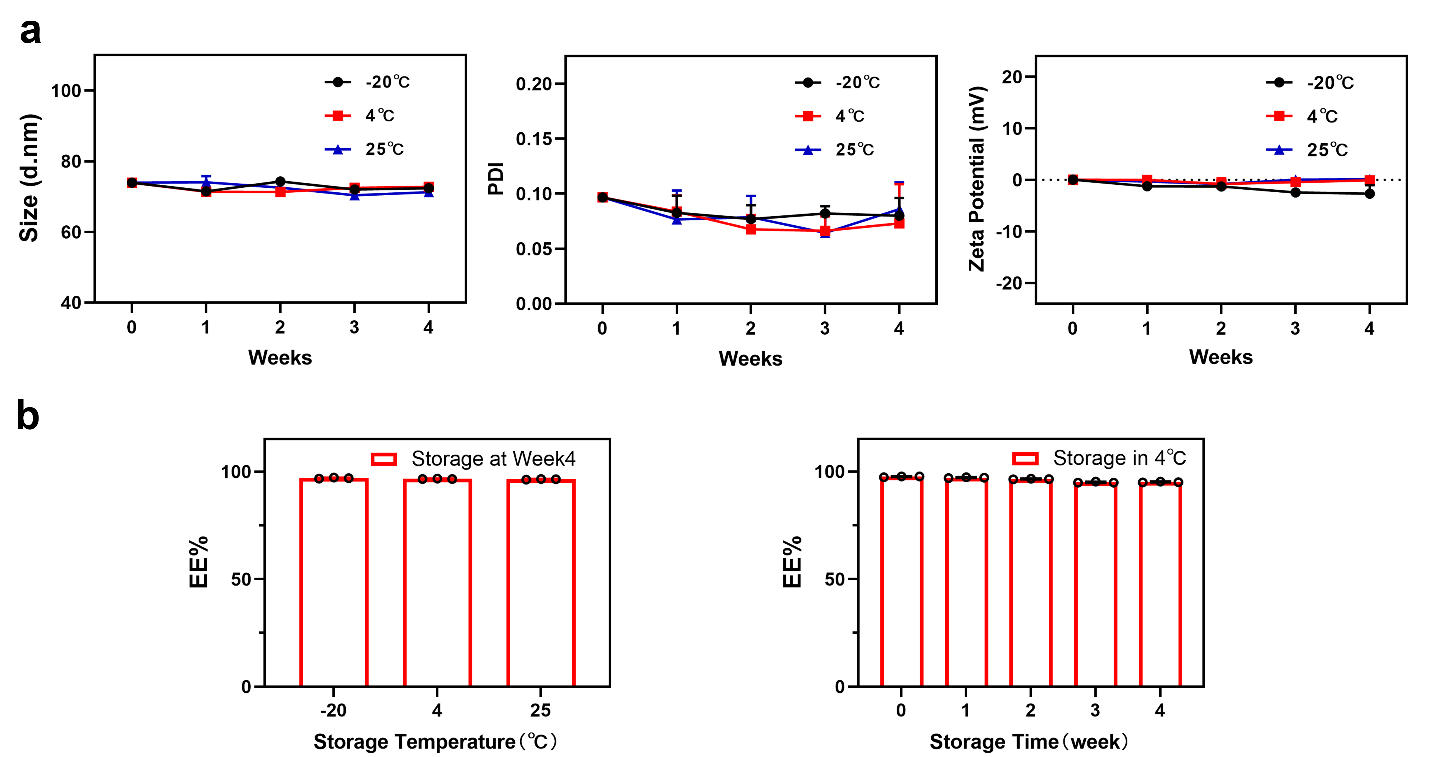


**Supplemental Fig.S8 The stability of siRNA encapsulating tLNP.** (a) The siRNA encapsulating tLNPs were stored at -20 ℃, 4 ℃ and 25 ℃ for 1 week to 4 weeks, then the sizes, polydispersity index (PDI) and ζ potential were determined. (b) The encapsulation efficiency (EE%) of siRNA encapsulating tLNP that stored at -20 ℃, 4 ℃ and 25 ℃ for 4 weeks or at 4 ℃ for 1 week to 4 weeks were determined. Data were shown as means ± SDs (n = 3).

Figure. S9.


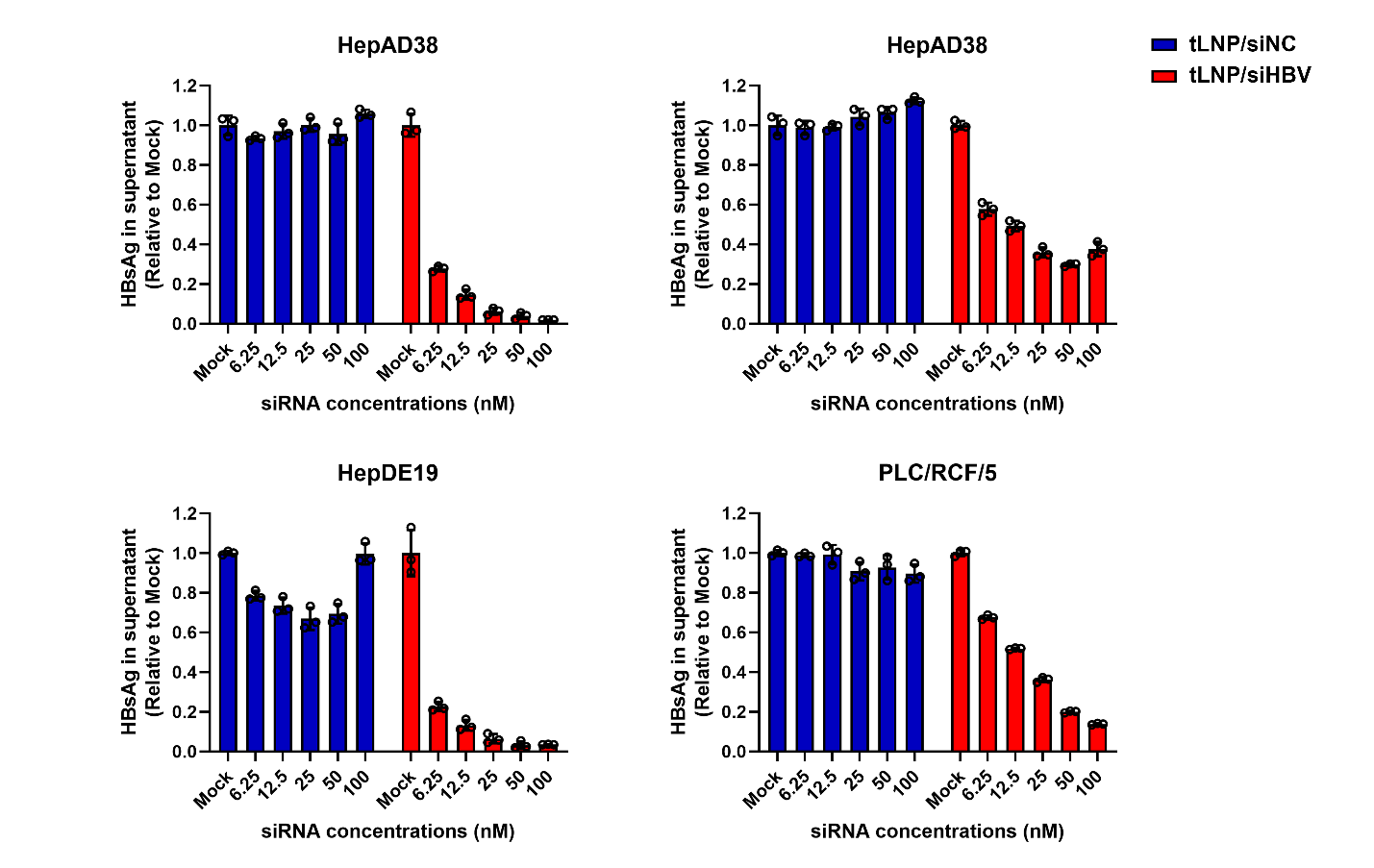


**Supplemental Fig.S9 Anti-HBV efficacy of tLNP/siHBV in HBV cell models.** HepAD38, HepDE19 and PLC/RCF/5 cells were transfected with different concentrations of tLNP/siHBV (0, 6.25, 12.5, 25, 50 and 100 nM) for 48 h, and the HBsAg and HBeAg levels in the supernatants were determined via ELISA kits. Data were shown as means ± SDs (n = 3).

Figure. S10.


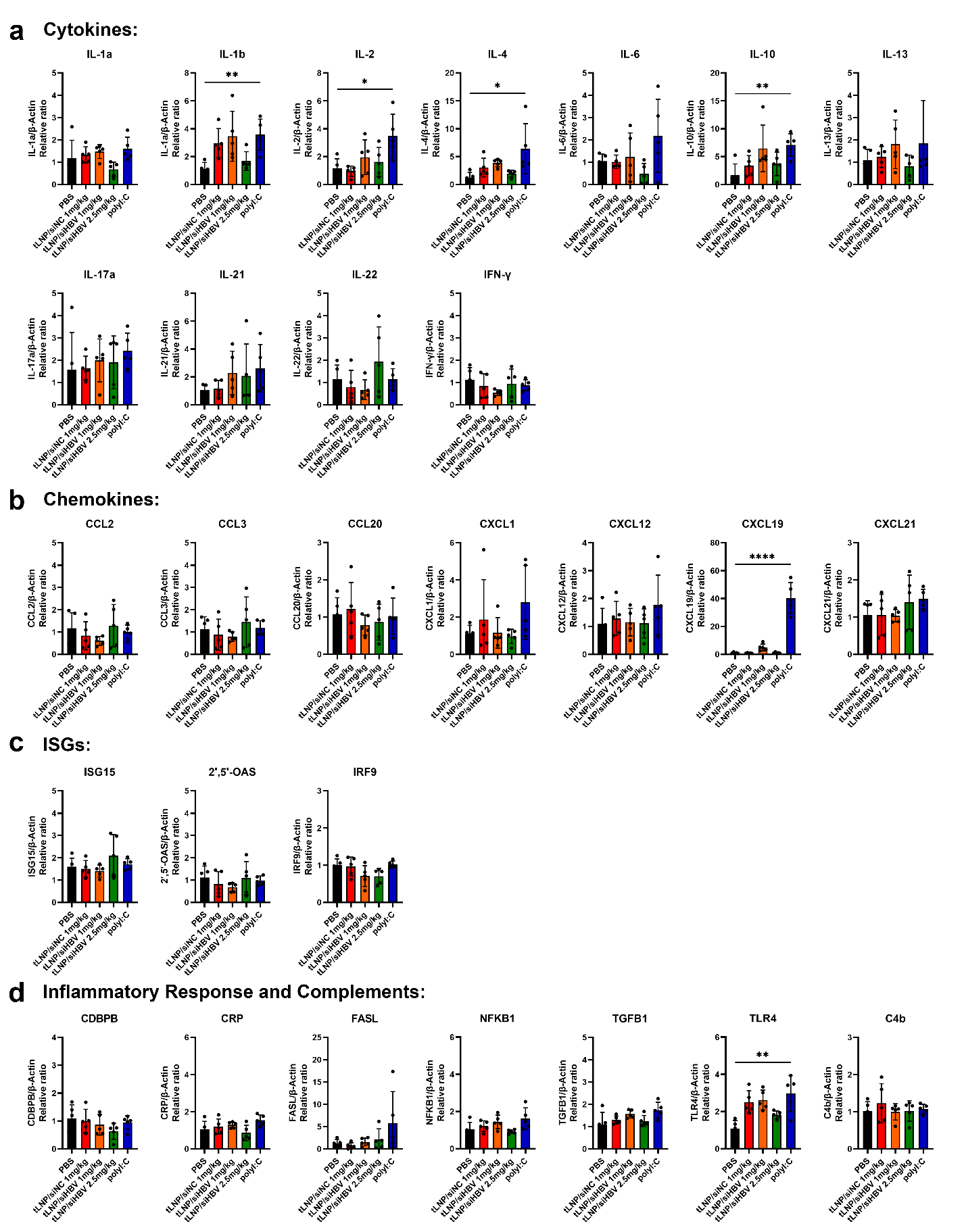


**Supplemental Fig.S10 The inflammatory responses of tLNP/siHBV in normal mice.** (a) C57BL/6 mice were treated with PBS, tLNP/siNC (1 mg/kg) or tLNP/siHBV (1 mg/kg or 2.5 mg/kg) via intravenous injection. Mice were sacrificed at 48 h post-treatment, intrahepatic mRNAs were extracted, reverse transcribed, and applied to RT-qPCR analysis with indicated primers. The relative expression levels of cytokines, (b) chemokines, (c) interferon-stimulated genes (ISGs) and (d) inflammatory response and complements-related genes were displayed in bar plots. Data were analyzed using unpaired two-way Student’s *t*-test analysis, and shown as means ± SDs (n = 5). **P*<0.05, ***P*<0.01, ****P*<0.001, and *****P*<0.0001.

Figure. S11.


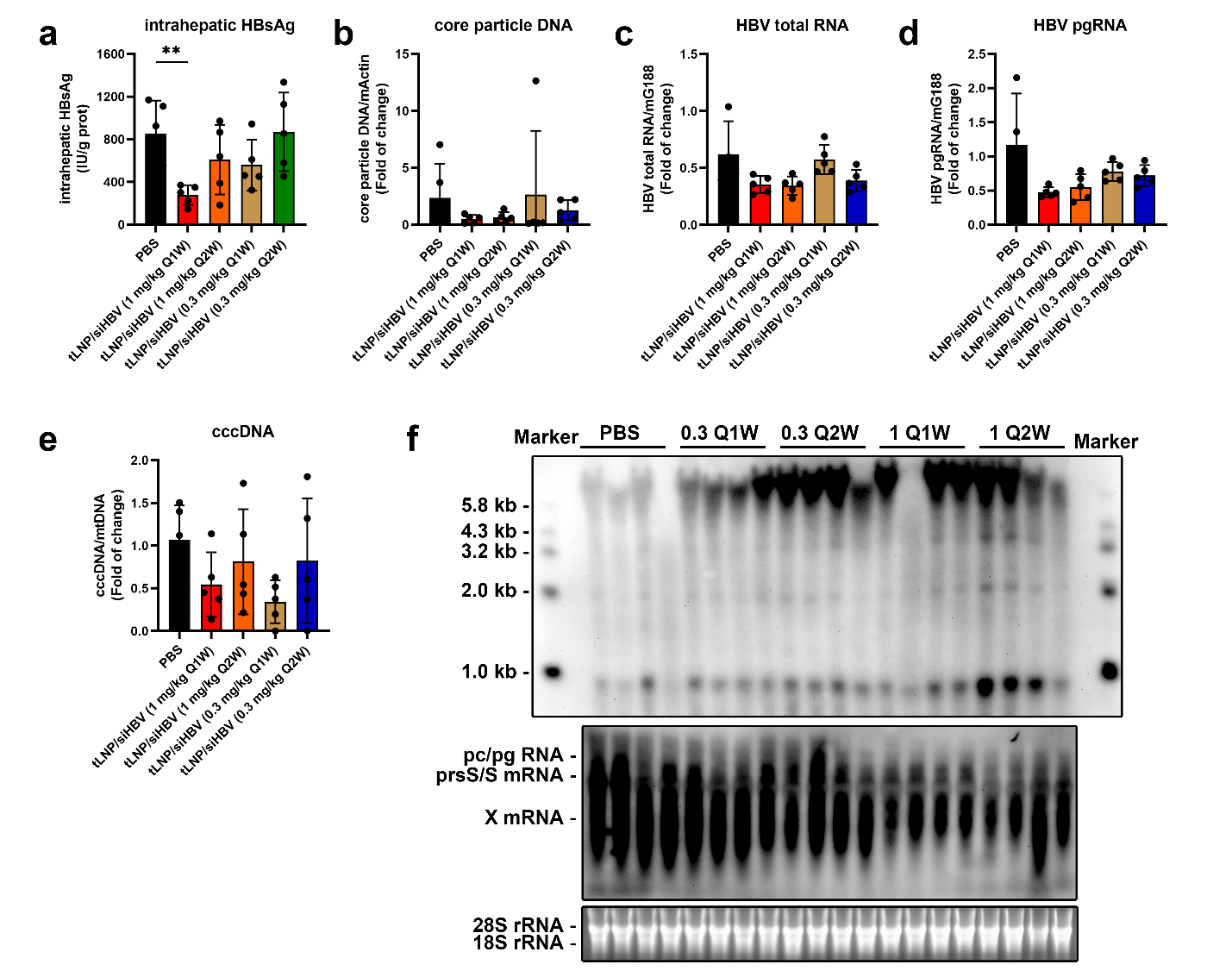


**Supplemental Fig.S11 Intrahepatic viral markers in rAAV-HBV1.3 mouse model after multi-dose treatment of tLNP/siHBV.** (a) Mice were treated with different doses (1 mg/kg, 0.3 mg/kg) of tLNP/siHBV at once weekly (Q1W) or once biweekly (Q2W) frequencies then stopped for three weeks before sacrificing. Liver was sampled and applied for relative detection. The intrahepatic HBsAg levels, (b) core particle DNA levels, (c) HBV total RNA and (d) pre-genomic (pg) RNA levels were determined relatively. Data were analyzed using unpaired two-way Student’s *t*-test analysis, and shown as means ± SDs (n = 4). **P*<0.05, ***P*<0.01, and ****P*<0.001. (e) The intrahepatic cccDNA levels were determined via RT-qPCR with specific primers and (f) via southern bolt analysis, and the intrahepatic HBV RNAs were also determined by northern blot analysis.

Figure. S12.


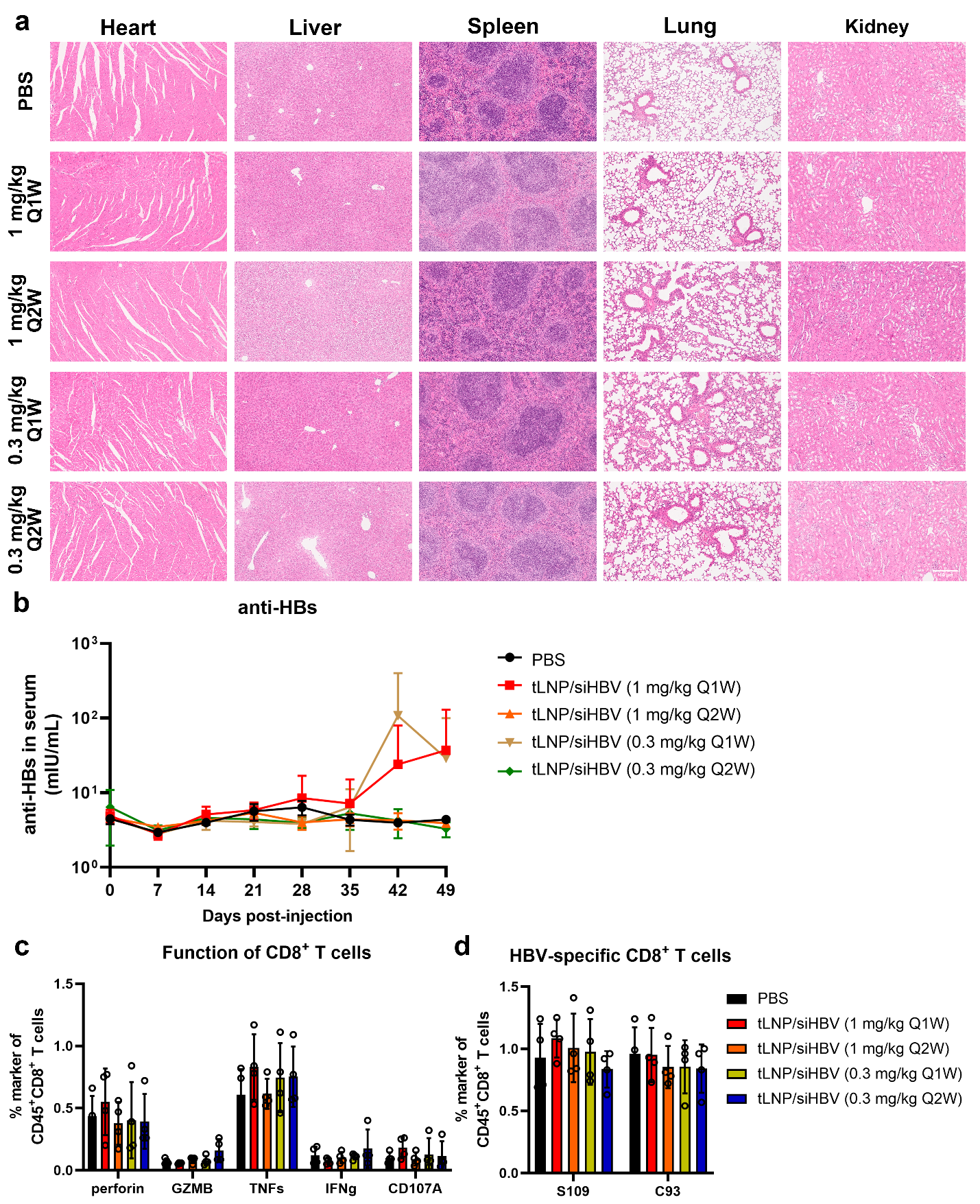


**Supplemental Fig.S12 Biosafety evaluation and immune responses of rAAV-HBV1.3 mouse model after multi-dose treatment of tLNP/siHBV.** (a) Histological analysis of heart, liver, spleen, lung and kidney of model mice after receiving multi-dose of tLNP/siHBV treatment. Scale bar indicated 100 μm. (b) The seral anti-HBs antibody levels were determined by commercial ELISA kits. Data were shown as means ± SDs (n = 8). (c) The function of CD8^+^ T cells and (d) the frequency of HBsAg-specific and core-specific CD8^+^ T cells in peripheral blood mononuclear cell (PBMC) were determined by flow cytometry analysis. Data were shown as means ± SDs (n = 4).

Figure. S13.


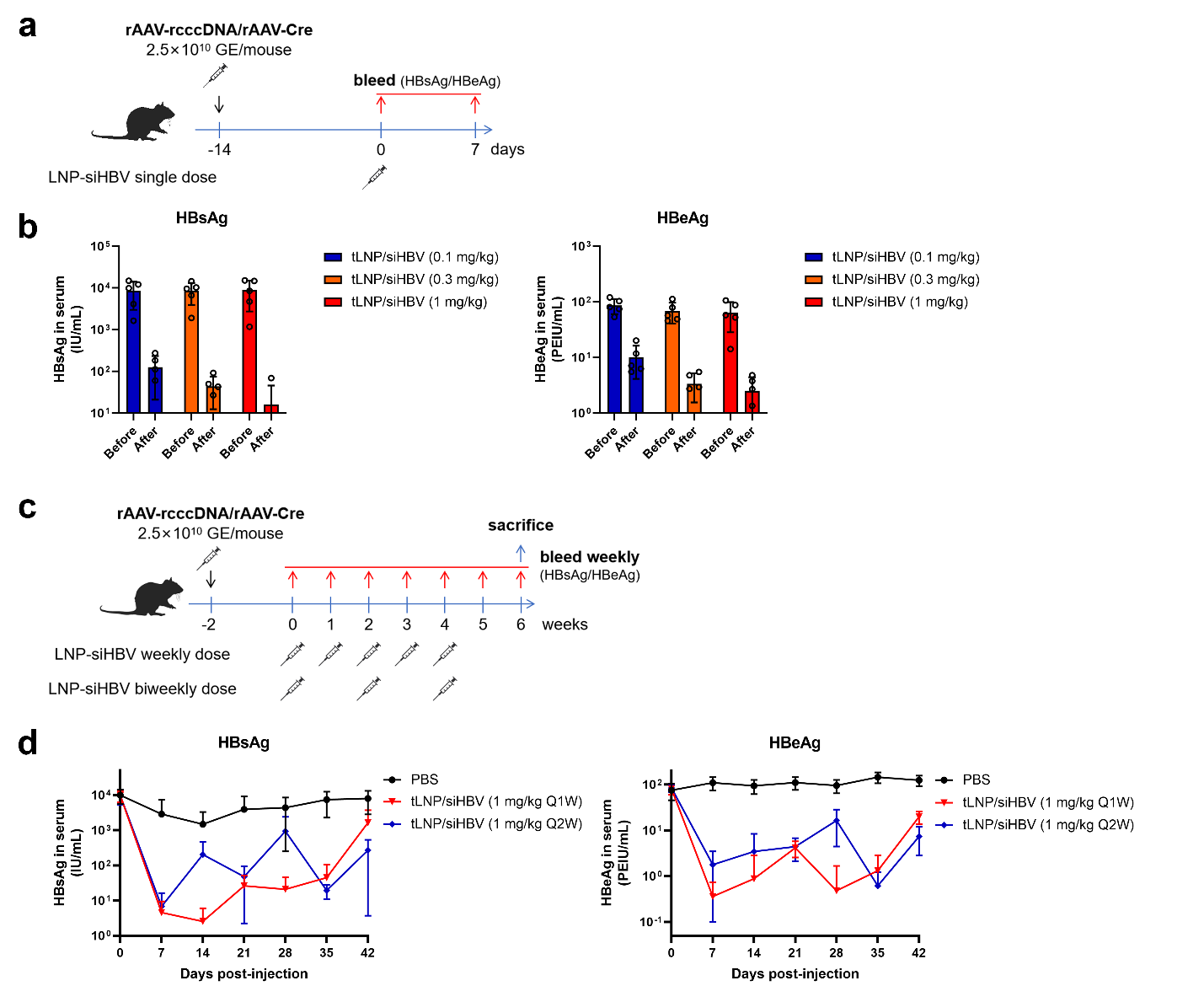


**Supplemental Fig.S13 Anti-HBV activity of tLNP/siHBV at single-dose or multi-dose frequencies in rAAV-rcccDNA/rAAV-Cre mouse model.** (a) Treatment and sampling schedule of the single dose study. Mice were injected with equal amounts of rAAV-rcccDNA and rAAV-Cre (2.5×10^10^ viral genome (v.g.) per mice), 2~4 weeks later, the mice were grouped according to seral HBsAg levels, and treated with tLNP/siHBV of different doses (0.1, 0.3 and 1 mg/kg). (b) The seral HBsAg and HBeAg levels were determined before treatment and after 7 days post-treatment. Data were shown as means ± SDs (n = 5). (c) Treatment and sampling schedule of the multi-dose study. The rAAV-rcccDNA/rAAV-Cre transduced mice were grouped according to seral HBsAg levels. Mice were then treated with tLNP/siHBV at the dose of 1 mg/kg, and at the frequency of once weekly (Q1W) or once bi-weekly (Q2W) for five weeks duration, then observed for another three weeks after drug withdrawal. (d) Mice were bled once weekly, and the seral levels of HBsAg and HBeAg were determined via commercial kits. Data were shown as means ± SDs (n = 6).

Figure. S14.


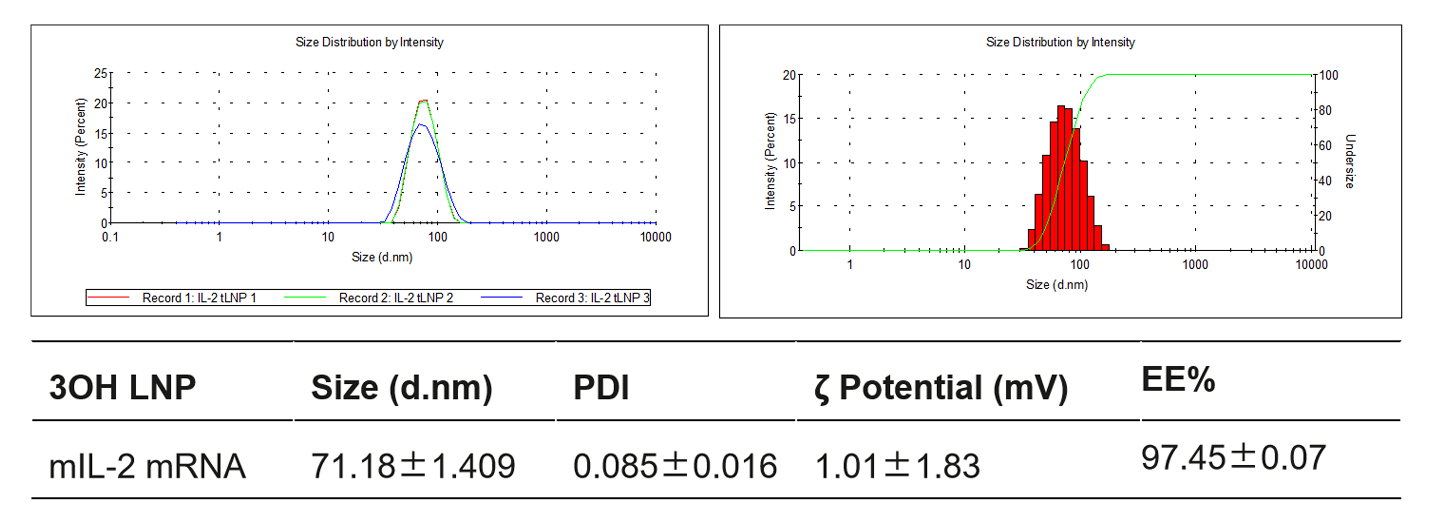


**Supplemental Fig.S14 The construction and evaluation of tLNP/IL2.** The mIL-2 mRNA was encapsulated within tLNP at the mass ratio of 1:20, and the size, polydispersity intensity (PDI), ζ potential and encapsulating efficiency (%) were determined.

Figure. S15.


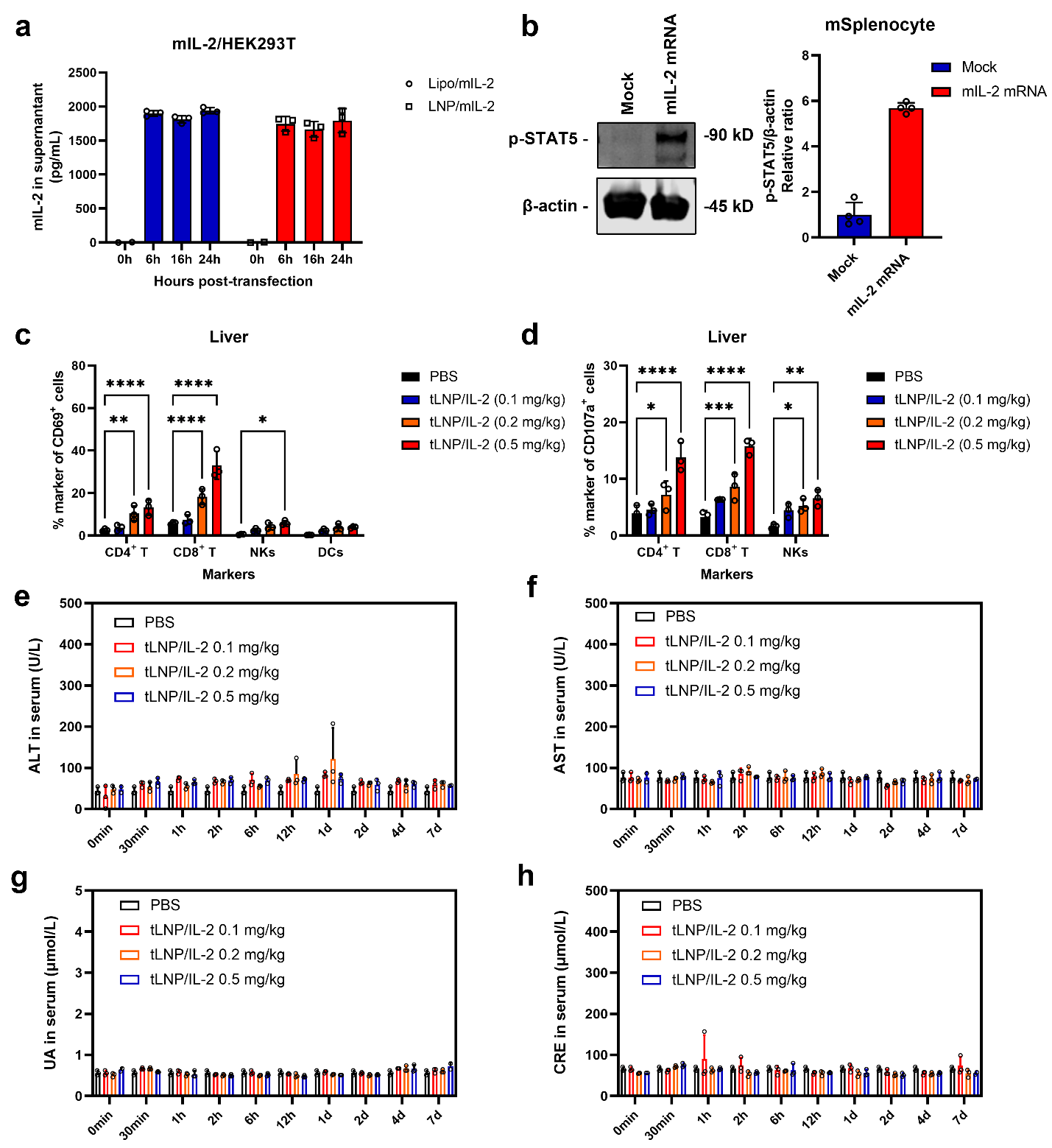


**Supplemental Fig.S15 The construction and examination of tLNP/IL2.** (a) Mouse IL-2 (mIL-2) mRNA was constructed and transfected with transfection reagent or encapsulated within tLNP in HEK293T cells, the expression levels of mIL-2 protein were determined by commercial ELISA kits at 6, 16 and 24 h post-transfection. Data were shown as means ± SDs (n = 3). (b) The supernatants were collected, applied to treat mouse splenocytes (mSplenocyte) and the phosphorylation levels of STAT5 were determined via western bolt analysis. Statistical analysis were shown as means ± SDs (n = 4). (c) mIL-2 mRNA encapsulating tLNP were intravenously injected into C57BL/6 mice at the doses of 0.1, 0.2 and 0.5 mg/kg. Mice were sacrificed at 24 h post-injection, and liver-associated lymphocytes were applied for surface CD69 and (d) CD107a staining and applied for flow cytometry analysis. The percentage of CD69-positive and CD107a-positive immune cells were calculated. Data were analyzed using two-way ANOVA with Sidak multiple comparison correction, and shown as means ± SDs (n = 3). **P*<0.05, ***P*<0.01, ****P*<0.001, and *****P*<0.0001. (e) Blood were sampled at indicated time points and were applied for ALT, (f) AST, (g) UA and (H) CRE levels determination via commercial kits. Data were shown as means ± SDs (n = 3).

Figure. S16.


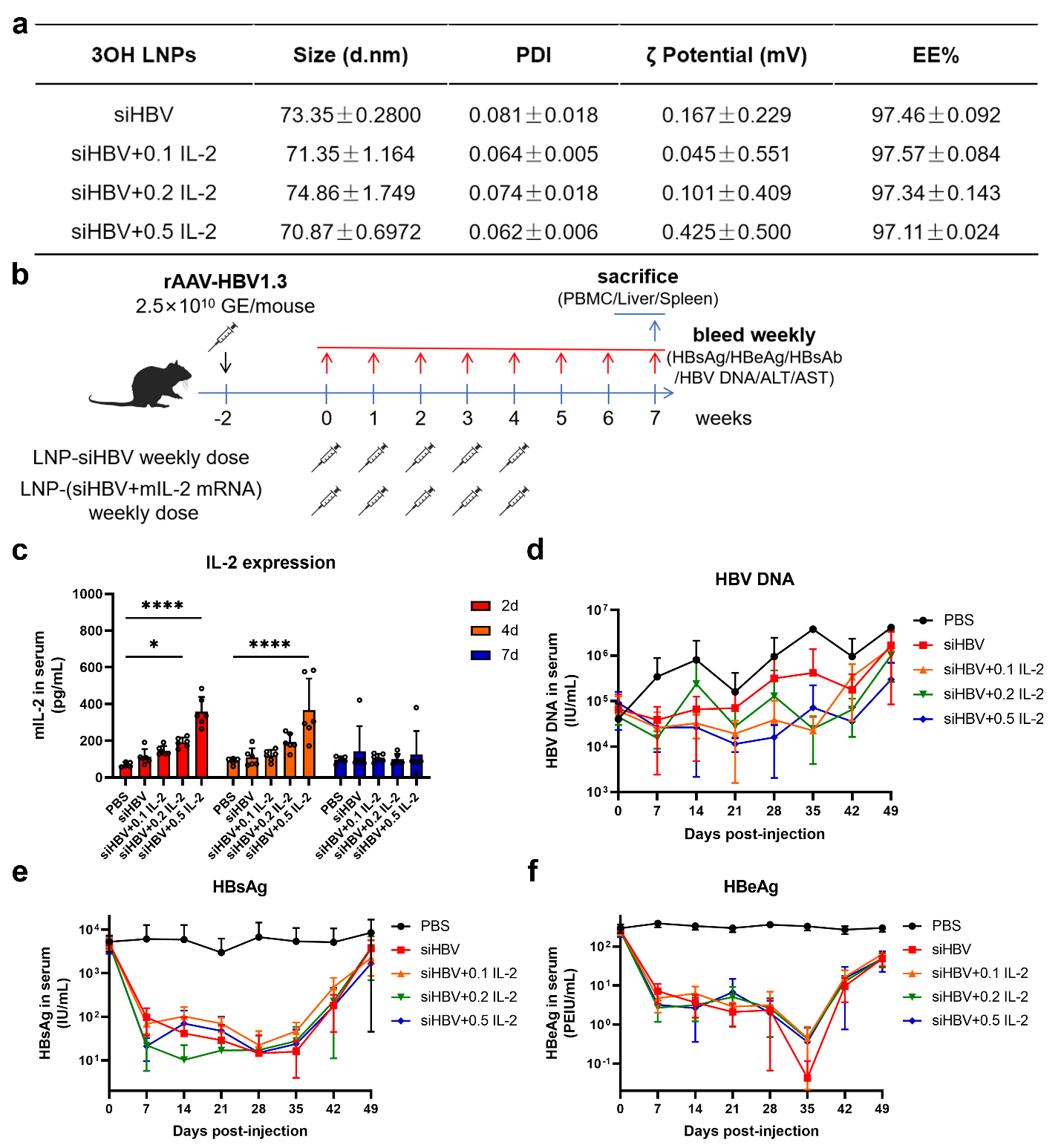


**Supplemental Fig.S16 Antiviral efficacy of tLNP/siHBVIL2 in rAAV-HBV1.3 mouse model.** (a) The mIL-2 mRNA was co-encapsulated with siHBV within a single tLNP formula at different mass ratios (10:1, 5:1 and 2:1), then the sizes, PDI, ζ potential and encapsulating efficiency (%) were determined. (b) Treatment and sampling schedule of combinational study. C57BL/6 mice of HBV replicating models were divided into groups and then intravenously injected with PBS, tLNP/siHBV and tLNP/siHBVIL2 at weekly dose frequency for five weeks then stopped for three weeks. (c) The expression levels of mIL-2 protein in multi-dose treated mice of HBV replication were determined by ELISA analysis at indicated time points. Data were analyzed using two-way ANOVA with Sidak multiple comparison correction, and shown as means ± SDs (n = 3). **P*<0.05, ***P*<0.01, ****P*<0.001, and *****P*<0.0001. (d) Mice were treated with PBS, siHBV with or without mIL-2 mRNA encapsulating tLNPs for five weekly doses then stopped for another three weeks. Blood were sampled once weekly, and seral HBV DNA levels, (e) HBsAg levels, and (F) HBeAg levels were determined via commercial kits. Data were shown as means ± SDs (n = 7~8).

Figure. S17.


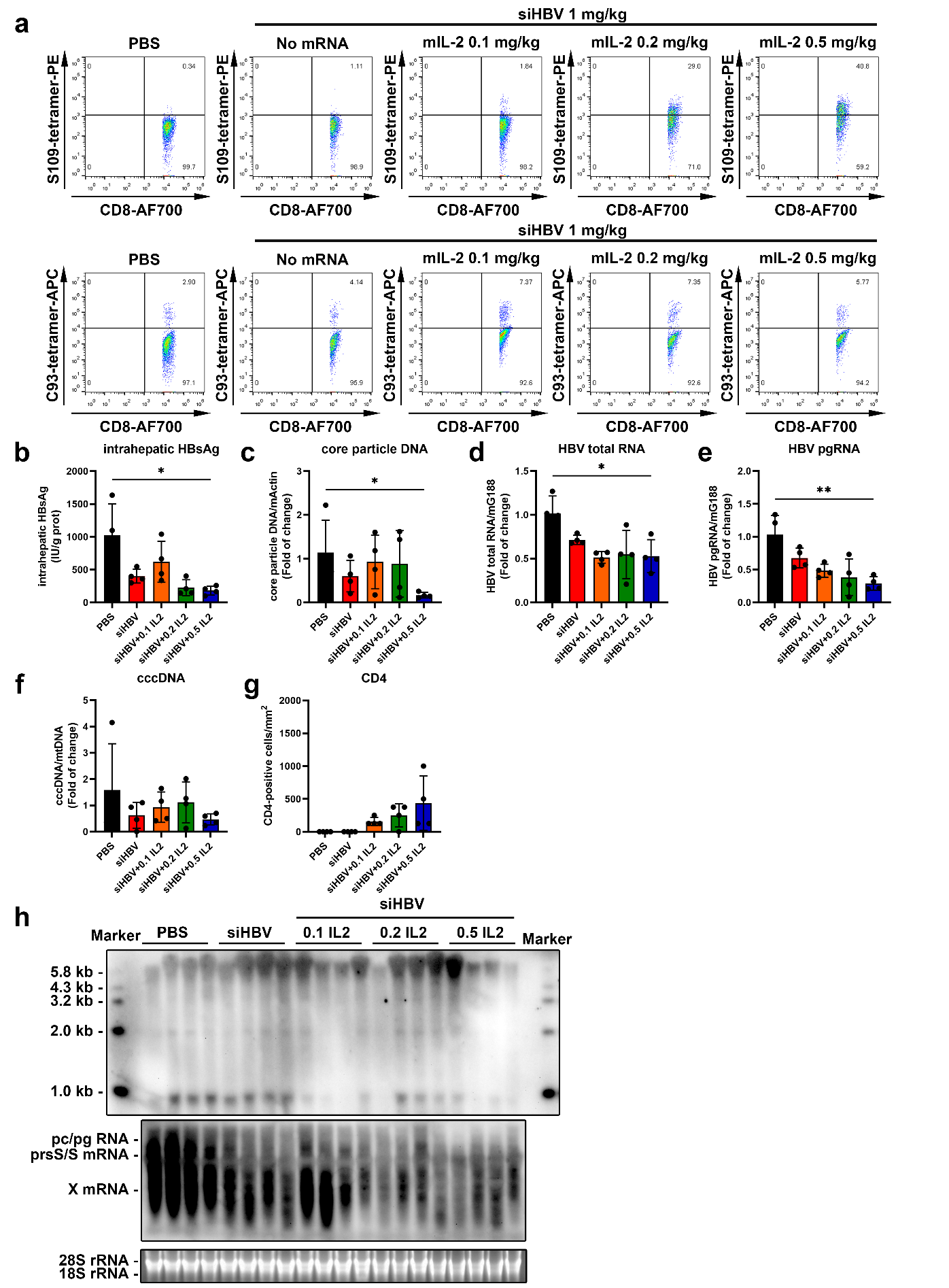


**Supplemental Fig.S17** **Intrahepatic viral markers in rAAV-HBV1.3 mouse model after multi-dose treatment of tLNP/siHBV and tLNP/siHBVIL2.** (a) Mice were treated with tLNP/siHBV (1 mg/kg) or tLNP/siHBV at different doses (siHBV:1 mg/kg, mIL-2 mRNA: 0.1, 0.2 and 0.5 mg/kg) at once weekly frequencies for five weeks, then stopped for three weeks before sacrificing. Liver-associated lymphocytes were collected and analyzed. Representative density plots of the proportion of HBsAg-specific (S109-tetramer-stained) and core-specific (C93-tetramer-stained) CD8^+^ T cells in gated CD3^+^ T cells. (b) The intrahepatic HBsAg levels, (c) core particle DNA levels, (d) HBV total RNA and (e) pgRNA levels were determined relatively. (f) The intrahepatic cccDNA levels were determined via RT-qPCR with specific primers. (g) The infiltration of CD4^-^ positive cells per mm^2^ liver sections was displayed. Data were analyzed using unpaired two-tailed Students’ *t*-test analysis, and shown as means ± SDs (n = 4). **P*<0.05, ***P*<0.01, ****P*<0.001, and *****P*<0.0001. (h) The intrahepatic cccDNA and HBV RNA levels were determined via southern bolt analysis and northern blot analysis, respectively.

Figure. S18.


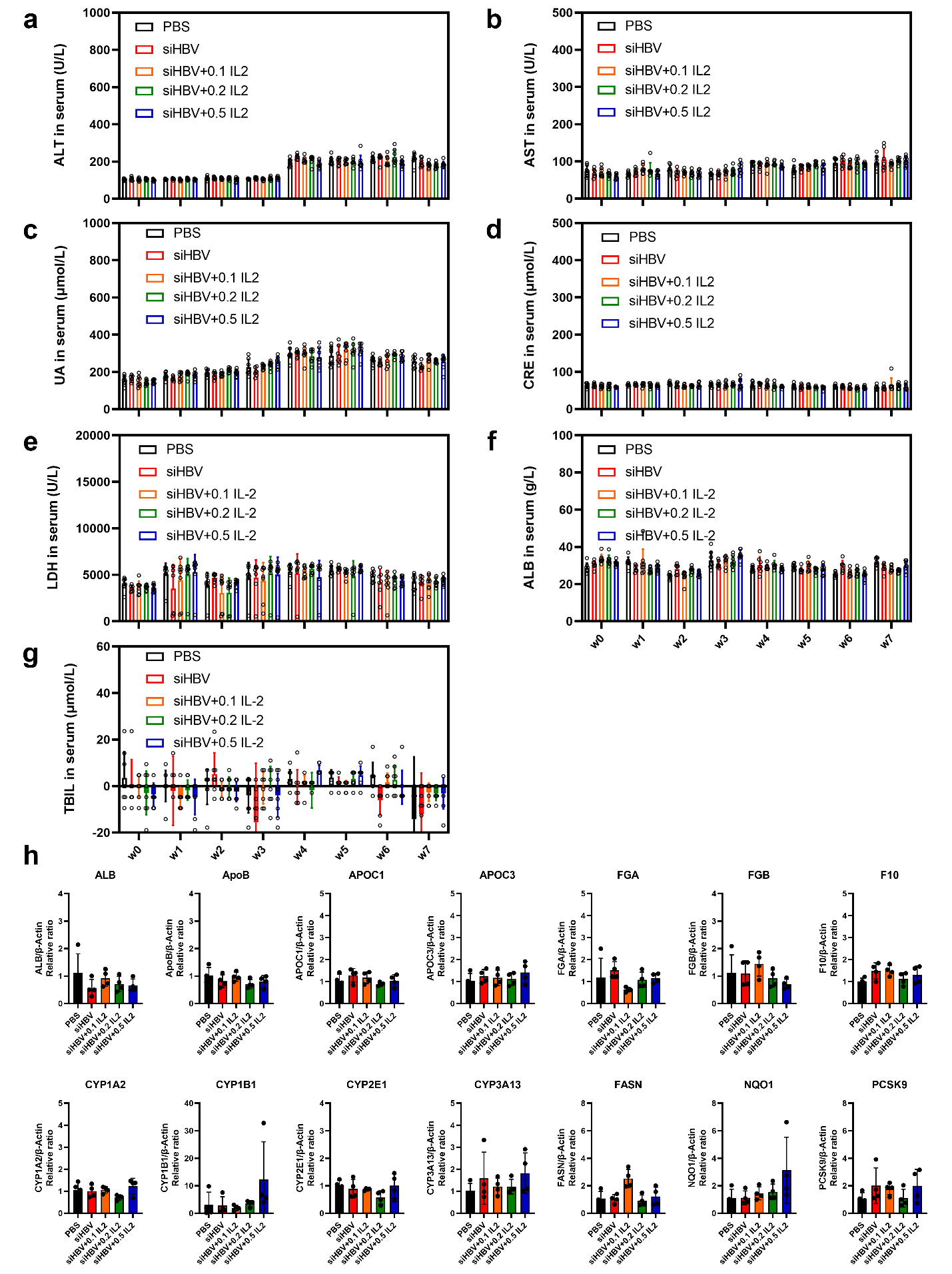


**Supplemental Fig.S18 Safety analysis of rAAV-HBV1.3 mouse model after multi-dose treatment of tLNP/siHBV and tLNP/siHBVIL2.** (a) Mice were treated with five doses of tLNP/siHBV or tLNP/siHBVIL2 at indicated doses, then stopped for three weeks. Blood were sampled once a week and were applied for ALT, (b) AST, (c) UA, (d) CRE, (e) LDH, (f) ALB, and (g) TBIL levels determination via commercial kits. Data were shown as means ± SDs (n = 7~8). (h) Intrahepatic mRNA was extracted, reverse transcribed, and applied to RT-qPCR analysis with specific primers. Expression levels of genes related to PBS control were displayed in bar plots. Data were shown as means ± SDs (n = 4).

Figure. S19.


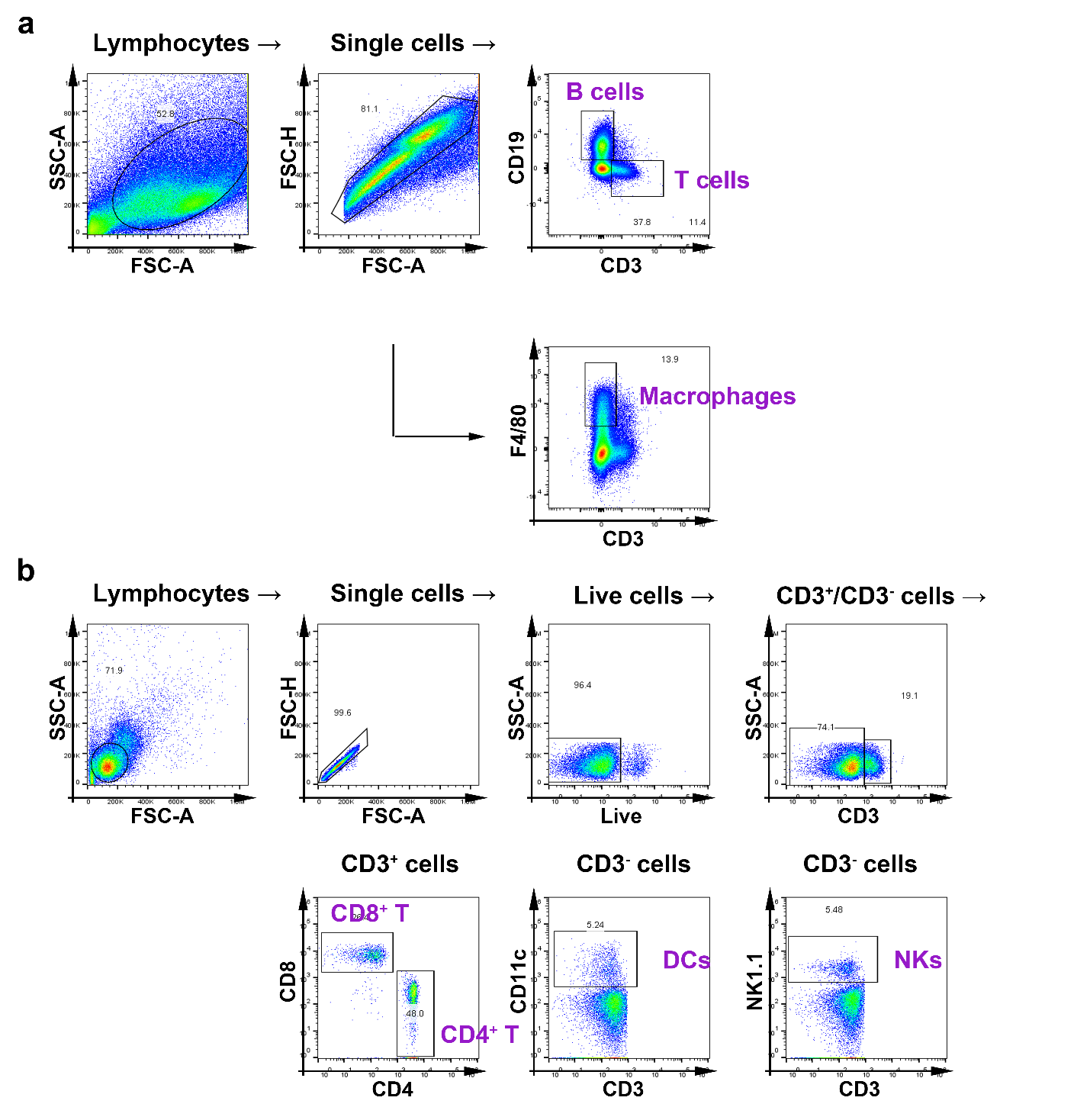


**Supplemental Fig.S9 Gating strategies for flow cytometry.** (a) Lymphocytes populations were defined using forward-scattered-light-area (FSC-A) and side-scattered-light-area (SSC-A) parameters. Doublets were excluded from lymphocytes populations by plotting FSC-height (FSC-H) against area (FSC-A) and gating on the diagonal population. Gating strategy for flow cytometric analysis of CD19^+^CD3^-^ B cells, CD3^+^CD19^-^ T cells, and F4/80^+^CD3^-^ macrophages. (b) Gating strategy for flow cytometric analysis of CD8^+^CD3^+^ T cells, CD4^+^CD3^+^ T cells, CD11c^+^CD3^-^ DCs and NK1.1^+^CD3^-^ NKs. Dead cells were excluded from analysis by gating on fixable viability dye (FVD-eFluor780-A)-negative cells.**Table S1.**

**Highly conserved motifs across HBV genomes predicted by MEME**

| **Motifs** | **Sequence** |
| --- | --- |
| MEME-1 | GGCTCAGTTTACTAGTGCCATTTGTTCAGTGGTTCGTAGGGCTTTCCCCC |
| MEME-2 | CGCGGGACGTCCTTTGTYTACGTCCCGTCGGCGCTGAATCCCGCGGACGA |
| MEME-3 | GAAGAAGAACTCCCTCGCCTCGCAGACGAAGGTCTCAATCGCCGCGTCGC |
| MEME-4 | TTCCTCTTCATCCTGCTGCTATGCCTCATCTTCTTGTTGGTTCTTCTGGA |
| MEME-5 | ACTGTTCAAGCCTCCAAGCTGTGCCTTGGGTGGCTTTGGGGCATGGACAT |
| MEME-6 | CCTCACAATACCRCAGAGTCTAGACTCGTGGTGGACTTCTCTCAATTTTC |
| MEME-7 | CTCCCCGTCTGTGCCTTCTCATCTGCCGGACCGTGTGCACTTCGCTTCAC |
| MEME-8 | CATCAGGATTCCTAGGACCCCTGCTCGTGTTACAGGCGGGGTTTTTCTTG |
| MEME-9 | GTCCTCCAATTTGTCCTGGYTATCGCTGGATGTGTCTGCGGCGTTTTATC |
| MEME-10 | AGACCACCAAATGCCCCTATCTTATCAACACTTCCGGAAACTACTGTTGT |
| MEME-11 | GGCTTTCAGTTATATGGATGATGTGGTATTGGGGGCCAAGTCTGTACAAC |
| MEME-12 | GCCTCATTTTGTGGGTCACCATATTCTTGGGAACAAGAGCTACAGCATGG |
| MEME-13 | TGCTGTACAAAACCTTCGGACGGAAACTGCACCTGTATTCCCATCCCATC |
| MEME-14 | CTCCTCTGCCGATCCATACTGCGGAACTCCTAGCCGCTTGTTTTGCTCGC |
| MEME-15 | GTGTCTTGGCCAAAATTCGCAGTCCCCAACCTCCAATCACTCACCAACCT |
| MEME-16 | TCCTGGGCTTTCGCAAAATTCCTATGGGAGTGGGCCTCAGTCCGTTTCTC |
| MEME-17 | AATGTTAGTATTCCTTGGACTCATAAGGTGGGAAACTTTACTGGGCTTTA |
| MEME-18 | GGTCTGTGCCAAGTGTTTGCTGACGCAACCCCCACTGGCTGGGGCTTGGC |
| MEME-19 | TCCCTTTTTACCGCTGTTACCAATTTTCTTTTGTCTTTGGGTATACATTT |
| MEME-20 | TATAAAGAATTTGGAGCTTCTGTGGAGTTACTCTCTTTTTTGCCTTCTGA |
| MEME-21 | CAAGGTATGTTGCCCGTTTGTCCTCTAATTCCAGGATCATCAACCACCAG |
| MEME-22 | GGCATACTTCAAAGACTGTGTGTTTAAAGACTGGGAGGAGTTGGGGGAGG |

Table S2.

**Target sites for HBV siRNA triggers**

| **siRNA** | **Sense sequence** | **Targeting RNA** | **Class** |
| --- | --- | --- | --- |
| si-220 | 5'-GACAAGAAUCCUCACAAUAdTdT-3' | pgRNA, PreS/S RNA | I |
| si-255 | 5'-CGUGGUGGACUUCUCUCAAdTdT-3' | pgRNA, PreS/S RNA | I |
| si-439 | 5'-GGUUCUUCUGGACUAUCAAdTdT-3' | pgRNA, PreS/S RNA | I |
| si-469 | 5'-CGUUUGUCCUCUAAUUCCAdTdT-3' | pgRNA, PreS/S RNA | I |
| si-675 | 5'-GUUUACUAGUGCCAUUUGUdTdT-3' | pgRNA, PreS/S RNA | I |
| si-726 | 5'-GGCUUUCAGUUAUAUGGAUdTdT-3' | pgRNA, PreS/S RNA | I |
| si-980 | 5'-GGAAAGUAUGUCAACGAAUdTdT-3' | pgRNA, PreS/S RNA | I |
| si-981 | 5'-GAAAGUAUGUCAACGAAUUdTdT-3' | pgRNA, PreS/S RNA | I |
| si-1260 | 5'-GCCGAUCCAUACUGCGGAAdTdT-3' | pgRNA, PreS/S RNA | I |
| si-1606 | 5'-GCAUGGAGACCACCGUGAAdTdT-3' | pgRNA, PreS/S, X RNA | II |
| si-1848 | 5'-GUUCAUGUCCUACUGUUCAdTdT-3' | pgRNA, PreS/S, X RNA | II |
| si-1857 | 5'-CUACUGUUCAAGCCUCCAAdTdT-3' | pgRNA, PreS/S, X RNA | II |
| si-1903 | 5'-GGACAUCGACCCUUAUAAAdTdT-3' | pgRNA, PreS/S, X RNA | II |
| si-1912 | 5'-CCCUUAUAAAGAAUUUGGAdTdT-3' | pgRNA, PreS/S, X RNA | II |
| si-2303 | 5'-CCAAAUGCCCCUAUCCUAUdTdT-3' | pgRNA | III |
| si-2330 | 5'-CCGGAGACUACUGUUGUUAdTdT-3' | pgRNA | III |
| si-2332 | 5'-GGAGACUACUGUUGUUAGAdTdT-3' | pgRNA | III |
| si-2390 | 5'-CGCAGACGAAGGUCUCAAUdTdT-3' | pgRNA | III |
| si-2413 | 5'-GCGUCGCAGAAGAUCUCAAdTdT-3' | pgRNA | III |
| si-2831 | 5'-GGAACAAGAUCUACAGCAUdTdT-3' | pgRNA | III |

Table S3.

**The conservativity of siRNA triggers in HBV sequences**

| **siRNA** | **19-mer coverage of HBV sequences** | **17-mer coverage of HBV sequences** |
| --- | --- | --- |
| si-220 | 63.45% (7,097/11,185) | 63.76% (7,131/11,185) |
| si-255 | 94.14% (10,530/11,185) | 98.42% (11,008/11,185) |
| si-439 | 51.01% (5,705/11,185) | 52.10% (5,827/11,185) |
| si-469 | 51.77% (5.791/11,185) | 51.93% (5,808/11,185) |
| si-675 | 90.43% (10,115/11,185) | 91.01% (10,179/11,185) |
| si-726 | 77.24% (8,639/11,185) | 77.56% (8,675/11,185) |
| si-980 | 24.37% (2,726/11,185) | 24.39% (2,728/11,185) |
| si-981 | 23.95% (2,679/11,185) | 24.39% (2,728/11,185) |
| si-1260 | 91.42% (10,225/11,185) | 92.96% (10,398/11,185) |
| si-1606 | 79.32% (8,872/11,185) | 79.77% (8,922/11,185) |
| si-1848 | 71.94% (8,047/11,185) | 72.00% (8,053/11,185) |
| si-1857 | 71.95% (8,048/11,185) | 72.21% (8,077/11,185) |
| si-1903 | 2.825% (316/11,185) | 2.834% (317/11,185) |
| si-1912 | 32.01% (3,580/11,185) | 37.66% (4,212/11,185) |
| si-2303 | 6.285% (703/11,185) | 6.688% (748/11,185) |
| si-2330 | 13.71% (1,534/11,185) | 13.72% (1,535/11,185) |
| si-2332 | 13.72% (1,535/11,185) | 13.80% (1,543/11,185) |
| si-2390 | 74.04% (8,281/11,185) | 74.30% (8,311/11,185) |
| si-2413 | 90.02% (10,069/11,185) | 90.36% (10,107/11,185) |
| si-2831 | 21.26% (2,378/11,185) | 21.31% (2,384/11,185) |
| si74 | 71.49% (7,996/11,185) | 85.86% (9,604/11,185) |
| si77 | 82.22% (9,196/11,185) | 86.03% (9,623/11,185) |

Table S4.

**The coverage of combined siRNA in HBV sequences**

| **siRNA** | **19-mer coverage of HBV sequences** | **17-mer coverage of HBV sequences** | **Targeting RNA** |
| --- | --- | --- | --- |
| si-675+si-726 | 94.55% (10,575/11,185) | 94.77% (10,600/11,185) | pgRNA, PreS/S RNA, intHBV |
| si-675+si-1260 | 98.69% (11,039/11,185) | 99.02% (11,075/11,185) | pgRNA, PreS/S RNA, intHBV |
| si-675+si-1606 | 98.26% (10,991/11,185) | 98.54% (11,022/11,185) | pgRNA, PreS/S, X RNA, intHBV |
| si-675+si-1848 | 97.41% (10,895/11,185) | 97.61% (10,918/11,185) | pgRNA, PreS/S, X RNA, intHBV |
| si-675+si-2413 | 98.48% (11,015/11,185) | 98.54% (11,022/11,185) | pgRNA, PreS/S RNA, intHBV |
| si-726+si-1260 | 98.19% (10,982/11,185) | 98.47% (11,014/11,185) | pgRNA, PreS/S RNA, intHBV |
| si-726+si-1606 | 94.74% (10,597/11,185) | 95.00% (10,626/11,185) | pgRNA, PreS/S, X RNA, intHBV |
| si-726+si-1848 | 87.47% (9,783/11,185) | 87.71% (9,810/11,185) | pgRNA, PreS/S, X RNA, intHBV |
| si-726+si-2413 | 96.71% (10,817/11,185) | 96.75% (10,821/11,185) | pgRNA, PreS/S RNA, intHBV |
| si-1260+si-1606 | 98.03% (10,965/11,185) | 98.37% (11,003/11,185) | pgRNA, PreS/S, X RNA, intHBV |
| si-1260+si-1848 | 98.55% (11,023/11,185) | 98.80% (11,051/11,185) | pgRNA, PreS/S, X RNA, intHBV |
| si-1260+si-2413 | 98.98% (11,071/11,185) | 99.13% (11,088/11,185) | pgRNA, PreS/S RNA, intHBV |
| si74+si77 | 89.82% (10,047/11,185) | 90.65% (10,139/11,185) | pgRNA, PreS/S, X RNA |

Table S5.

**Chemical modification of siRNA**

| **CM-siRNA** | **siRNA Sequences (5’→3’)** |
| --- | --- |
| si1260 | 5'-GCCGAUCCAUACUGCGGAAdTdT-3' |
|  | 5'-UUCCGCAGUAUGGAUCGGCdTdT-3' |
| si1848 | 5'-GUUCAUGUCCUACUGUUCAdTdT-3' |
|  | 5'-UGAACAGUAGGACAUGAACdTdT-3' |
| si1260-mm | 5'-GCmCmGAUmCmCmAUmACmUmGCmGGAAdTdT-3' |
|  | 5'-UmUmCmCmGCmAGUmAUmGGAUmCmGGCmdTdT-3' |
| si1848-mm | 5'-GUmUmCmAUmGUmCmCmUmACmUmGUmUmCmAdTdT-3' |
|  | 5'-UmGAACmAGUmAGGACmAUmGAACmdTdT-3' |
| si1260-Um | 5'-GCmCmGAUmCmCmAUmACmUmGCmGGAAdTdT-3' |
|  | 5'-UmUmCCGCAGUmAUmGGAUmCGGCdTdT-3' |
| si1848-Um | 5'-GUmUmCmAUmGUmCmCmUmACmUmGUmUmCmAdTdT-3' |
|  | 5'-UmGAACAGUmAGGACAUmGAACdTdT-3' |
| si1260-2m | 5'-GCmCmGAUmCmCmAUmACmUmGCmGGAAdTdT-3' |
|  | 5'-UUCCGCAGUmAUGGAUmCGGCdTdT-3' |
| si1848-2m | 5'-GUmUmCmAUmGUmCmCmUmACmUmGUmUmCmAdTdT-3' |
|  | 5'-UGAACAGUmAGGACAUmGAACdTdT-3' |
| si1260-Uf | 5'-GCmCmGAUmCmCmAUmACmUmGCmGGAAdTdT-3' |
|  | 5'-UfUfCCGCAGUfAUfGGAUfCGGCdTdT-3' |
| si1848-Uf | 5'-GUmUmCmAUmGUmCmCmUmACmUmGUmUmCmAdTdT-3' |
|  | 5'-UfGAACAGUfAGGACAUfGAACdTdT-3' |
| si1260-2f | 5'-GCmCmGAUmCmCmAUmACmUmGCmGGAAdTdT-3' |
|  | 5'-UUCCGCAGUfAUGGAUfCGGCdTdT-3' |
| si1848-2f | 5'-GUmUmCmAUmGUmCmCmUmACmUmGUmUmCmAdTdT-3' |
|  | 5'-UGAACAGUfAGGACAUfGAACdTdT-3' |
| si1260-am | 5'-GCmCGmAUmCCmAUmACmUGmCGmGAmAdTdT-3' |
|  | 5'-UmUCmCGmCAmGUmAUmGGmAUmCGmGCmdTdT-3' |
| si1848-am | 5'-GUmUCmAUmGUmCCmUAmCUmGUmUCmAdTdT-3' |
|  | 5'-UmGAmACmAGmUAmGGmACmAUmGAmACmdTdT-3' |
| si1260-amf | 5'-GfCmCfGmAfUmCfCmAfUmAfCmUfGmCfGmGfAmAfdTdT-3' |
|  | 5'-UmUfCmCfGmCfAmGfUmAfUmGfGmAfUmCfGmGfCmdTdT-3' |
| si1848-amf | 5'-GfUmUfCmAfUmGfUmCfCmUfAmCfUmGfUmUfCmAfdTdT-3' |
|  | 5'-UmGfAmAfCmAfGmUfAmGfGmAfCmAfUmGfAmAfCmdTdT-3' |
| si1260-aESC | 5'-CmsUmsGmCmCmGmAfUmCfCfAfUmAmCmUmGmCmGmGmAmAm-3' |
|  | 5'-UmsUfsCmCmGmCfAmGfUfAmUmGmGmAfUmCfGmGmCmAmGmsAmsGm-3' |
| si1848-aESC | 5'-UmsUmsGmUmUmCmAfUmGfUfCfCmUmAmCmUmGmUmUmCmAm-3' |
|  | 5'-UmsGfsAmAmCmAfGmUfAfGmGmAmCmAfUmGfAmAmCmAmAmsGmsAm-3' |
| si1260-GalNac | 5'-CmsUmsGmCmCmGmAfUmCfCfAfUmAmCmUmGmCmGmGmAmAm-(GalNac)_3_-3' |
|  | 5'-UmsUfsCmCmGmCfAmGfUfAmUmGmGmAfUmCfGmGmCmAmGmsAmsGm-3' |
| si1848-GalNac | 5'-UmsUmsGmUmUmCmAfUmGfUfCfCmUmAmCmUmGmUmUmCmAm-(GalNac)_3_-3' |
|  | 5'-UmsGfsAmAmCmAfGmUfAfGmGmAmCmAfUmGfAmAmCmAmAmsGmsAm-3' |
| si1260-2m(PS) | 5'-GCmCmGAUmCmCmAUmACmUmGCmGGAAdTsdT-3' |
|  | 5'-UUCCGCAGUmAUGGAUmCGGCdTsdT-3' |
| si1848-2m(PS) | 5'-GUmUmCmAUmGUmCmCmUmACmUmGUmUmCmAdTsdT-3' |
|  | 5'-UGAACAGUmAGGACAUmGAACdTsdT-3' |
| si1260-amf(PS) | 5'-GfCmCfGmAfUmCfCmAfUmAfCmUfGmCfGmGfAmAfdTsdT-3' |
|  | 5'-UmUfCmCfGmCfAmGfUmAfUmGfGmAfUmCfGmGfCmdTsdT-3' |
| si1848-amf(PS) | 5'-GfUmUfCmAfUmGfUmCfCmUfAmCfUmGfUmUfCmAfdTsdT-3' |
|  | 5'-UmGfAmAfCmAfGmUfAmGfGmAfCmAfUmGfAmAfCmdTsdT-3' |

**References**

1. Jones, L. J., Yue, S. T., Cheung, C. Y. & Singer, V. L. RNA quantitation by fluorescence-based solution assay: RiboGreen reagent characterization. *Anal. Biochem.* **265**, 368-374 (1998).

2. Wu, M. et al. A novel recombinant cccDNA-based mouse model with long term maintenance of rcccDNA and antigenemia. *Antiviral Res* **180**, 104826 (2020).

3. Zai, W. et al. Long-Term Hepatitis B Virus Infection Induces Cytopathic Effects in Primary Human Hepatocytes, and Can Be Partially Reversed by Antiviral Therapy. *Microbiol Spectr* **10**, e132821 (2022).

4. Li, G. et al. Recombinant covalently closed circular DNA of hepatitis B virus induces long-term viral persistence with chronic hepatitis in a mouse model. *Hepatology* **67**, 56-70 (2018).

5. Richner, J. M. et al. Modified mRNA Vaccines Protect against Zika Virus Infection. *Cell* **168**, 1114-1125 (2017).
