# Supplementary figures and images for: Optimized RNA interference therapeutics combined with interleukin-2 mRNA for treating hepatitis B virus infection
Source: Signal Transduct Target Ther. 2024 Jun 21;9:150. doi: 10.1038/s41392-024-01871-8 (PMC11189933; doi:10.1038/s41392-024-01871-8)

Original and uncropped films of Western blots


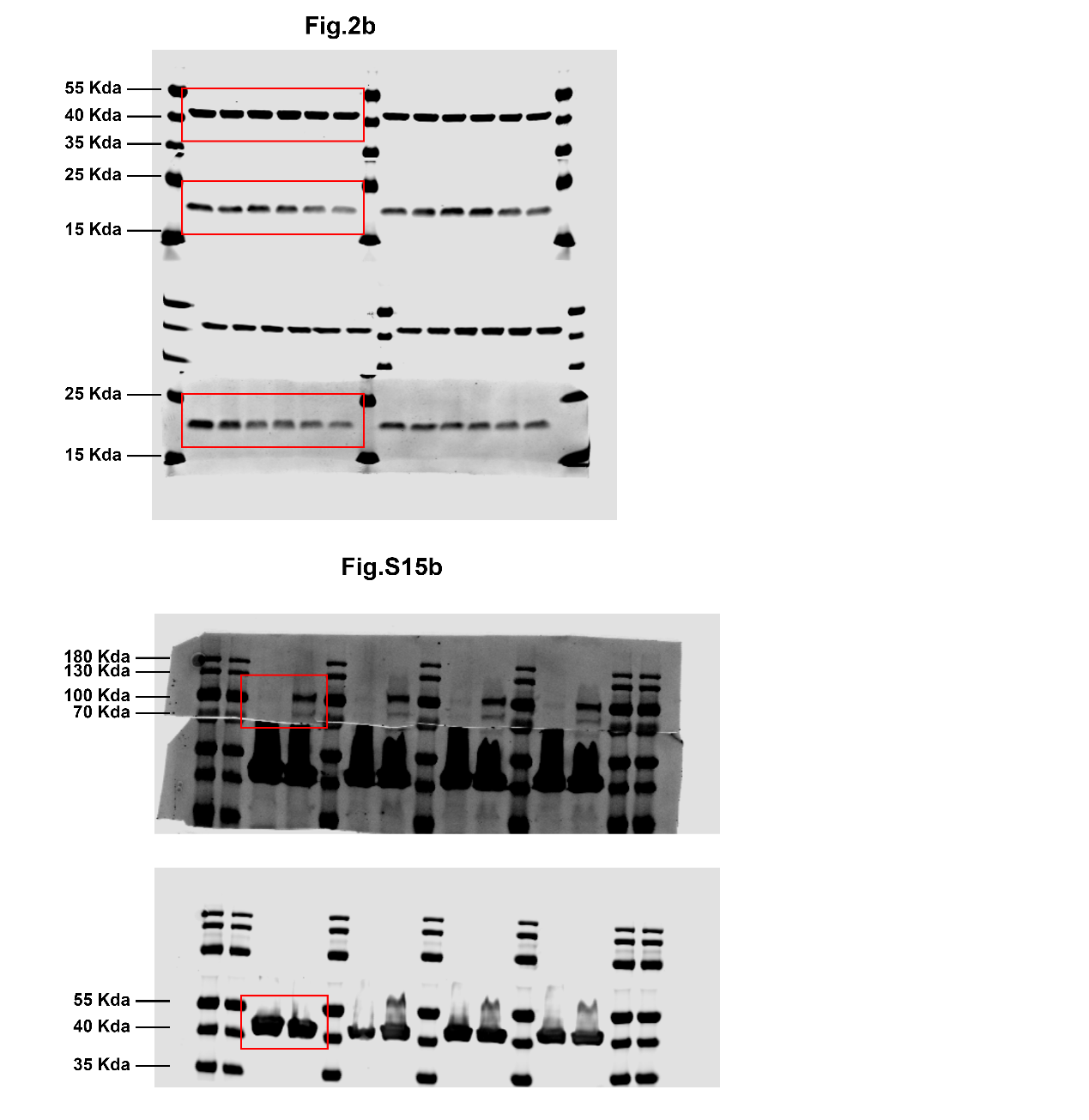

Supplement: Supplementary file 2 — Supplementary Materials [file 41392_2024_1871_MOESM2_ESM.docx]
